# Integrative genomic analysis in K562 chronic myelogenous leukemia cells reveals that proximal NCOR1 binding positively regulates genes that govern erythroid differentiation and Imatinib sensitivity

**DOI:** 10.1093/nar/gkv642

**Published:** 2015-06-27

**Authors:** Mark D. Long, Patrick R. van den Berg, James L. Russell, Prashant K. Singh, Sebastiano Battaglia, Moray J. Campbell

**Affiliations:** Department of Pharmacology and Therapeutics, Roswell Park Cancer Institute, Elm & Carlton Streets, Buffalo, NY 14263, USA

## Abstract

To define the functions of NCOR1 we developed an integrative analysis that combined ENCODE and NCI-60 data, followed by *in vitro* validation. NCOR1 and H3K9me3 ChIP-Seq, FAIRE-seq and DNA CpG methylation interactions were related to gene expression using bootstrapping approaches. Most NCOR1 combinations (24/44) were associated with significantly elevated level expression of protein coding genes and only very few combinations related to gene repression. DAVID's biological process annotation revealed that elevated gene expression was uniquely associated with acetylation and ETS binding. A matrix of gene and drug interactions built on NCI-60 data identified that Imatinib significantly targeted the NCOR1 governed transcriptome. Stable knockdown of NCOR1 in K562 cells slowed growth and significantly repressed genes associated with NCOR1 cistrome, again, with the GO terms acetylation and ETS binding, and significantly dampened sensitivity to Imatinib-induced erythroid differentiation. Mining public microarray data revealed that NCOR1-targeted genes were significantly enriched in Imatinib response gene signatures in cell lines and chronic myelogenous leukemia (CML) patients. These approaches integrated cistrome, transcriptome and drug sensitivity relationships to reveal that NCOR1 function is surprisingly most associated with elevated gene expression, and that these targets, both in CML cell lines and patients, associate with sensitivity to Imatinib.

## INTRODUCTION

Nuclear receptor corepressor 1 (NCOR1) and its paralog NCOR2/SMRT play prominent roles in controlling the epigenome in health and disease. These proteins were discovered as a result of their interactions with nuclear receptors, for example thyroid hormone and retinoic acid receptors (1,2), and subsequently were shown to interact with a wider array of transcription factors (TFs) (reviewed in (3–5)). Therefore, it is not surprising that NCOR1 and NCOR2/SMRT are both essential for development and homeostasis (6–8). Also these proteins are distorted in many cancers through altered expression levels (9–21), splice variants (22,23), mutation status (24,25) and genetic variation (26).

Classically, NCOR1 and NCOR2/SMRT are considered to be transcriptional corepressors that sustain and drive repressive epigenetic environments wherever they interact with TFs (27,28). For example, at the sites of nuclear receptor binding within gene enhancer regions, NCOR1 recruits histone deacetylase proteins, namely HDAC3 (2) to maintain elevated H3K9me3 levels and either limit or silence transcription (29,30). Repressive histone marks also act as platforms to induce DNA CpG methylation (reviewed in (31)), for example as seen with the vitamin D receptor (VDR) (32). Furthermore, increased corepressor binding also promotes direct association with the transcriptional repressor ZBTB33/KAISO (33) and targets increased DNA methylation (33–35). More recently, NCOR2/SMRT binding to SPEN/SHARP (36) has been shown to be important for gene silencing mediated by Xist (37).

Set against this literature on the corepressor function of NCOR1 and NCOR2/SMRT, a number of studies have revealed roles for these proteins to behave in a manner that suggests they can act as positive regulators of gene expression. For example, relatively quickly after their identification, it was revealed that corepressors could enhance expression of genes that were repressed (38,39). More recently, NCOR2/SMRT has been shown in breast cancer cells to act as a coactivator for p53 (40) and ERα (41).

This incomplete understanding of NCOR1 and NCOR2/SMRT function may arise for a number of reasons, including specificity of function and experimental design (42). In part, it also reflects biases introduced by studying NCOR1 function in the context of candidate gene loci. The genome-wide distribution of NCOR1 binding sites, the so-called cistrome, has not been analyzed comprehensively in human cells (43,44), although murine studies have been undertaken (45). Therefore the genome-wide distribution and specificity of TF interactions and associations with gene expression have not been comprehensively investigated.

A large volume of data has become publically available to address this knowledge gap as a result of the efforts of consortia such as ENCODE (46–49), as well as other functional genomics investigators (50–56). Collectively, these studies have begun to reveal considerable insight into the structure and regulation of the human genome. These studies have demonstrated a hitherto unsuspected complexity in terms of the variation and diversity in many key steps in the control of transcription including: the extent of the genome that is transcribed, the distribution of TF binding across the human genome, the functional differences in the spatial relationships between proximal and distal binding, the interplay between TFs and different co-regulating partners, the number of functionally different RNA molecules that are transcribed and the impact of mechanisms that process and edit RNA molecules. Although the biological meaning of these findings is not without debate (57,58), these efforts have catalyzed further investigations, and a re-appraisal of TF function.

We therefore exploited various genomic data sets to investigate NCOR1 function. ENCODE undertook ChIP-Seq toward NCOR1 in K562 cells, which are a chronic myelogenous leukemia (CML) cell line (59), that resemble erythrocyte precursors (60). These cells also harbor the BCR-ABL translocation, also known as the Philadelphia Chromosome (61,62), which forms a chimeric protein that in turn is a target for the kinase specific inhibitor Imatinib (63). The targeting of this protein with Imatinib is one of the key success stories in so-called targeted cancer therapies and provides a paradigm for precision medicine in general (64). The cells have been extensively investigated by ENCODE investigators with ∼600 different genomic data sets publically available.

**Figure 1.**
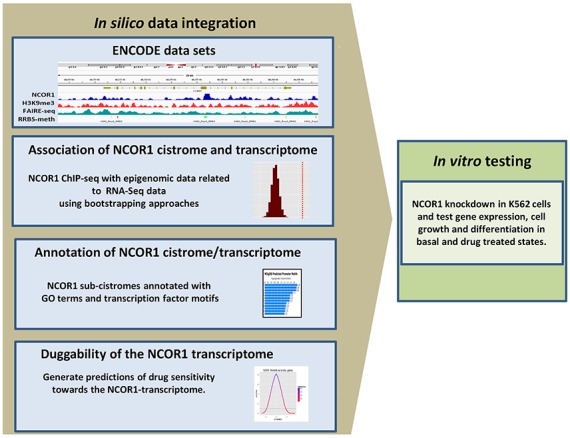
*In silico* and *in vitro* approaches to interrogate the NCOR1 cistrome in K562 cells. Workflow used to define and analyze the NCOR cistrome. NCOR1 and H3K9me3 ChIP-Seq, FAIRE-Seq, RRBS-methylation and RNA-Seq data were integrated to evaluate the association of NCOR1 with gene expression in K562 cells. The expression of genes associated with NCOR1 binding was investigated with bootstrapping approaches and the drug sensitivity of these gene groups were used to parse NCI-60 drug sensitivity data. The gene expression and drug sensitivity predictions arising from these analyses were tested *in vitro*.

The genomic distribution of NCOR1 was overlaid with different epigenetic states and gene expression patterns. Specifically, we combined NCOR1 ChIP-Seq with other data sets that measured chromatin status (FAIRE-seq), histone patterns (H3K9me3) and DNA methylation (RRBS). To overcome the limitations of studying of a single cell line, we applied a random sampling approach termed bootstrapping to simulate data for comparison to experimentally observed findings (65). In this manner, the different combinations of these epigenomic data were related to gene expression (RNA-seq) using bootstrap approaches. To annotate these cistrome-transcriptome relationships further we exploited pharmacogenomic data from the NCI-60 database (66) to investigate drug sensitivities toward the NCOR1 cistrome-associated genes. These data integration approaches generated predictions concerning the impact of NCOR1 binding on gene expression, drug responses and cell fate status that were subsequently tested *in vitro* (Figure 1).

Together these *in silico* and *in vitro* approaches revealed two principal findings; namely NCOR1 binding in a proximal position to gene transcriptional start sites is predominantly associated with elevated gene expression; and that the NCOR1-dependent transcriptome is significantly associated with regulating sensitivity toward Imatinib.

## MATERIALS AND METHODS

### Materials and cells

K562 cells were cultured in RPMI 1640 (Invitrogen) containing 10% fetal bovine serum and supplemented with streptomycin (5 U/ml) and penicillin (5 μg/ml) at 37°C with 5% CO_2_. Imatinib and Nilotinib were obtained from Sigma.

### Cell proliferation assay

Proliferation was measured by counting K562 cells every 24 h using automated Vi-Cell^TM^ cell viability analyzer (Beckman Coulter). Cells were seeded in 5 ml of media 5000 cells/well. Each experiment was performed in triplicate wells in biological triplicate experiments. Statistical analysis was carried out using two-tailed Student's *t*-test.

### Erythroid differentiation assay

Exponentially growing cells in medium were mixed 1:1 with the benzidine solution (benzidine dihydrochloride 2 mg/ml, 3% v/v acetic acid with 1% hydrogen peroxide freshly added) and photographed (10 X, Olympus DP80) after 5 min incubation. The blue oxidized form of benzidine is a marker of erythroid differentiation (67). The images were analyzed with *CellProfiler* (ver. 2.1.1) (68) to determine the proportion of benzidine-positive cells.

### Stable NCOR1 knockdown in K562 cells

Knockdown of NCOR1 in K562 cells was achieved by lentiviral shRNA constructs targeting *NCOR1* (Dharmacon, V2LHS_91777/V2LHS_91778) from RPCI shRNA Resource at Rowell Park Cancer Institute and selected with puromycin (2 μg/ml).

### Western immunoblotting for NCOR1

This was undertaken as described previously (21) using a NCOR1 antibody (A301–145A, Bethyl).

### Data analyses and integration

All analyses, unless otherwise indicated, were undertaken using the R platform for statistical computing (version 3.1.0) http://www.R-project.org/ (67,69) (and a range of library packages were implemented in Bioconductor (70) (Supplementary Table S1).

### ENCODE data sets

NCOR1 ChIP-Seq, H3K9me3 ChIP-Seq, c-Myc ChIP-Seq POL2 ChIP-Seq, CTCF ChIP-Seq H3K27me3 ChIP-Seq, RRBS, FAIRE-seq and RNA-seq data, all derived from K562 cells were developed through the ENCODE project and downloaded from the ENCODE project file repository through UCSC (Supplementary Table S1). All analyses used the peaks annotated by the ENCODE consortium with the following modifications. Very large and low intensity NCOR1 ChIP-Seq peaks (>100 kB and signal value <2) were removed (Supplementary Figure S1). Duplicate methyl-RRBS-seq data were merged to determine average methylation levels of CpGs with at least 10 reads in both replicates. Whole cell long poly-A RNA-seq data were filtered (IDR < 0.1) and average replicate reads per kilobase per million mapped reads (RPKMs) were Box-Cox transformed using the car package. Only genes classified as protein coding (Ensembl biotype classification) were used for further analysis. Genes that passed irreproducible discovery rate (IDR) filtering but did not have RPKM values were considered as not expressed and tagged as ‘off’.

### Visualization of binding profiles around transcription start site

Average number of peaks around transcription start site (TSS) (±2 kb) region for ChIP-Seq experiments (NCOR, H3K9me2) and FAIRE-Seq were calculated using ChIPseeker using the getTagMatrix function. The number of reads was then extracted for the plot. Since the absolute number of peaks differs between the different sequencing experiment, data were normalized for visualization purposes. The average methylation levels across the TSS region were calculated by extracting the distance from the CpGs in the RRBS data set to the TSS with bedtools (http://bedtools.readthedocs.org/en/latest/—version 2.22.1) closest/closestBed function and the hg19 TSS file (UCSC). The data were then filtered to keep values +/− 1 kb from the TSS and the methylation values were fitted with a polynomial linear model for visualization in the plot. The final plot was generated in R using the plot() and lines() functions.

### Integration of NCOR1 cistrome, epigenome and transcriptome data

All the data sets annotated around the TSS regions were defined as +/− 1 kb for all Ensembl gene TSS locations. Each 2 kb TSS region was annotated with respect to ChIP-Seq, FAIRE-seq and RRBS-seq data sets by calculating the overlap between each peak and TSS region. Overlaps of the different data sets were examined using GenomicRanges and were deemed positive if at least 25% of the peak genomic region overlapped with the 2 kb TSS region. The methylation status of each TSS region was calculated as the average methylation of all detectable CpGs within the region and were defined as low (C_l_, < 30%) or high (C_h_, > 30%) methylation status.

Using the above parameters, if a TSS overlapped with a peak then it was flagged as True (t) and a TSS without any overlap was flagged as False (f). In this fashion, all genes were annotated with respect to the NCOR1 (N_true_ (N_t_), N_false_ (N_f_)), H3K9me3 (H_t_, H_f_), open and closed chromatin status (FAIRE-seq; F_t_, F_f_) and CpG methylation (C_l_, C_h_). TSS regions containing no CpGs with detectible methylation levels by above criteria were considered separately (C_f_). For example, N_t_H_f_C_l_F_t_ indicates a subset of genes with a TSS that contains both NCOR1 (N_t_) and FAIRE (F_t_) peaks, as well as low average CpG methylation levels (C_l_), while lacking H3K9me3 peaks within their TSS regions (H_f_). If a particular mark was not considered in the analysis, it was flagged ‘independent’ (N_i_, H_i_, C_i_, F_i_). For instance, N_t_H_t_C_i_F_i_ indicates the TSS state associated with a subset of genes containing both NCOR1 and H3K9me3 peaks while considering neither CpG methylation nor chromatin status.

Gene expression for each gene subset was determined using the whole cell long poly-A RNA-seq data. To test the significance of these values bootstrapping analysis (65) was used to determine if the average gene expression of the subsets differed from the average expression of 100 000 simulated signatures. Similarly, the proportion of observed and expected on/off genes for each subset was calculated using hypergeometric testing.

### Allocating NCOR1 into 100 bp bins to build a gene expression prediction model

ENCODE investigators previously developed a binning model to examine the relationships between histone marks and TF binding, and gene expression (71). This was done under the assumption that specific regions of DNA binding/enrichment highly correlate with expression at different sites across the gene body and promoter. Therefore the NCOR1 binding strength was determined across genes, by dividing each gene into 81 bins. First, from all protein coding genes, genes with a minimum size of 4100 bp were selected and divided into 40 × 100 bp bins centered on the TSS, 40 × 100 bp bins centered on the transcription termination site (TTS) and one bin for the remaining gene body. For each of the 81 bins, the binding of NCOR1 was determined using BigWigAverageOverBed (http://hgdownload.cse.ucsc.edu/admin/exe/) and binding signal was log-transformed. Correlation analysis between binding signal intensity and logged RNA-Seq RPKM values was run using the cor.test() function in R. Correlation coefficient and mean of the binding signal were then plotted in two different graphs with one value per each bin. Plots were generated using the ggplot2.

### TF motif enrichment analysis

To identify enrichment of TF motifs around the 5 kb TSS regions of genes bound by NCOR1 (N_t_ gene list) and the indicated subsets, the ENCODE ChIP-Seq Significance Tool (http://encodeqt.simple-encode.org/) was used to mine for significant enrichment within the genes of TFs listed in the ENCODE ChIP-Seq data derived in K562 cells. One-tailed hypergeometric test followed by Benjamini–Hochberg multiple hypothesis correction was applied to identify significant enrichment of TF binding. Common TFs across subsets were extracted, ranking data were normalized and hierarchical clustering using Euclidean distance was run to identify TF clusters. Lastly, a heatmap was generated to visualize the differential enrichment of factors between the conditions. In order to identify predicted and known motifs within the unique peaks in each subset, the Homer tool (72) was used with the findMotifsGenome.pl script.

### Functional annotation

Functional annotation was performed using the David suite of tools (http://david.abcc.ncifcrf.gov/home.jsp). The Functional Annotation Clustering tool was used to select enriched functional clusters and the resulting lists were used to parse out unique and common annotation terms.

### Identifying drug sensitivity associated with genes in the NCOR1 cistrome

The NCI-60 anticancer drug screen has established relative drug sensitivity and gene expression profiles across the NCI-60 cell lines which are available through CellMiner (73). From these data the mean centered (Z scores) drug sensitivity and gene expression profiles were extracted. For each compound (20 503 total drugs, including 108 FDA approved), the respective drug sensitivity profile was correlated (Pearson's *r*) to each gene (*n* = 26 062) and significant drug-gene correlations were compiled (FDR ≤ 0.1). Correlations between gene expression and drug sensitivity were both positive (high relative gene expression/high relative drug sensitivity, low relative gene expression/low relative drug sensitivity) and negative (low relative gene expression/high relative drug sensitivity, high relative gene expression/low relative drug sensitivity). In this manner, genes with expression patterns across the NCI-60 cell lines that were predictive of a given drugs sensitivity were determined for all available compounds. To reveal putative drugs whose sensitivity was regulated by NCOR1 modulation, all drug-associated gene sets were then examined for NCOR1 cistrome enrichment (hypergeometric test (FDR ≤ 0.01)). Only FDA approved compounds with significant NCOR1 cistrome enrichment by these criteria were considered for further analysis.

### Analyses of gene expression in K562 cells with knockdown of NCOR1 and following drug treatment

K562 cells stably transfected with either a vector control (K562-shCTR) or shRNA to *NCOR1* (K562-shNCOR1), treated for 48 h with either 500 nM Imatinib or DMSO, and total RNA isolated using the Trizol method. To measure global changes in mRNA, biological triplicates were analyzed using Illumina microarray (Illumina HT12v4). Data analyses were undertaken with the lumi and limma packages (74,75). Briefly, data from arrays were background corrected, transformed by the variance-stabilizing transformation method and normalized within and between arrays using the robust spline method. Probes with normalized intensities reaching significant detection (detection *P*-value < 0.05) in at least nine of 12 samples were retained for analysis, and differential expression between groups was determined using the lmFit and eBayes functions (FDR ≤ 0.05, fold change ≥ 1.3). Although the R code used in this manuscript is different from the one published on Biostar, S.B. would like to acknowledge Biostar (www.biostar.org) for the initial graphic idea for Supplementary Figure S7.

### Analyses of gene expression in K562 cells and CML patient samples

Publically available microarray data were exploited to examine enrichment of gene sets associated either with NCOR1 binding, modulated by NCOR1 knockdown, or affected by drug treatment. Data were downloaded directly as deposited in the Gene Expression Omnibus for analyses. Hypergeometric tests were applied to establish the extent and significance of overlap between the gene lists. Differentially expressed genes (DEGs) were determined in K562 cells upon treatment of Imatinib (1 uM, 24 h, GSE1922) and Nilotinib (0.05 uM, 24 h, GSE19567) and assessed for NCOR1 cistrome enrichment as described above. Similarly, DEGs (FC > 1.2, *P* < 0.05, as described in Bruennert *et al*.) from CD34+ blasts from CML patients treated with Imatinib for 7 days (400 mg daily) were examined for NCOR1 enrichment. Genes associated with resistance to Imatinib therapy were determined by analyses of primary CML patient data (GSE14671 (76)). This study included CD34+ blasts from patients treated with Imatinib and who had a significant response to therapy at 12 months (responders) compared to those where the BCR-ABL translocation remained detectable in a proportion of the blasts (non-responders). Gene expression from 59 patient samples (41 responders and 18 non-responders) were analyzed with SimpleAffy (77). Background correction (Robust Multi-array (RMA) method) followed by quantile normalization and removal of outliers generated the processed data from which DEGs were established using samr; 10 000 permutation testing was applied to identify genes that were significantly up-regulated or down-regulated (>1.5-fold) in the CML patients who displayed a complete response to Imatinib compared to those where only a partial response was observed. One hundred seventy-one genes were significantly different between the responders and non-responders.

### Circos plots of selected gene sets

ChIA-PET files for phospho-RNA-pol II containing inter- and intra-chromosomal interaction were downloaded from the ENCODE project file repository through UCSC as: wgEncodeGisChiaPetK562Pol2InteractionsRep1.bed and wgEncodeGisChiaPetK562Pol2InteractionsRep2.bed (Supplementary Table S1). Only intervals present in both files were taken into consideration for the analysis. Genomic intervals corresponding to genes significantly down- and up-regulated were compared to the ENCODE files using BEDTools. Overlaps were calculated for both sides of the chromosomal interactions and output data were used to generate Circos plots using the Circos tool (http://circos.ca).

## RESULTS

### The NCOR1 cistrome positively associates with elevated gene expression

To address the relationship between the NCOR1 cistrome distribution and gene expression, we exploited data in K562 cells. NCOR1 ChIP-Seq data were generated by ENCODE using the BroadPeak algorithm (78). An enrichment threshold of log2 signal intensity >1 was selected, to eliminate peaks that were of low intensity and very large size (greater than 100 kb) (Supplementary Figure S1). After this filtering, the NCOR1 ChIP-Seq data set consisted of 9053 peaks of an average genomic length of 4560 bp. From these, 2043 NCOR1 peaks were selected that were in a proximal relationship to all protein coding genes (Supplementary Figure S2). In this manner, NCOR1 peaks were aligned within ±1 kb of the TSS of 1899 protein coding genes.

**Figure 2.**
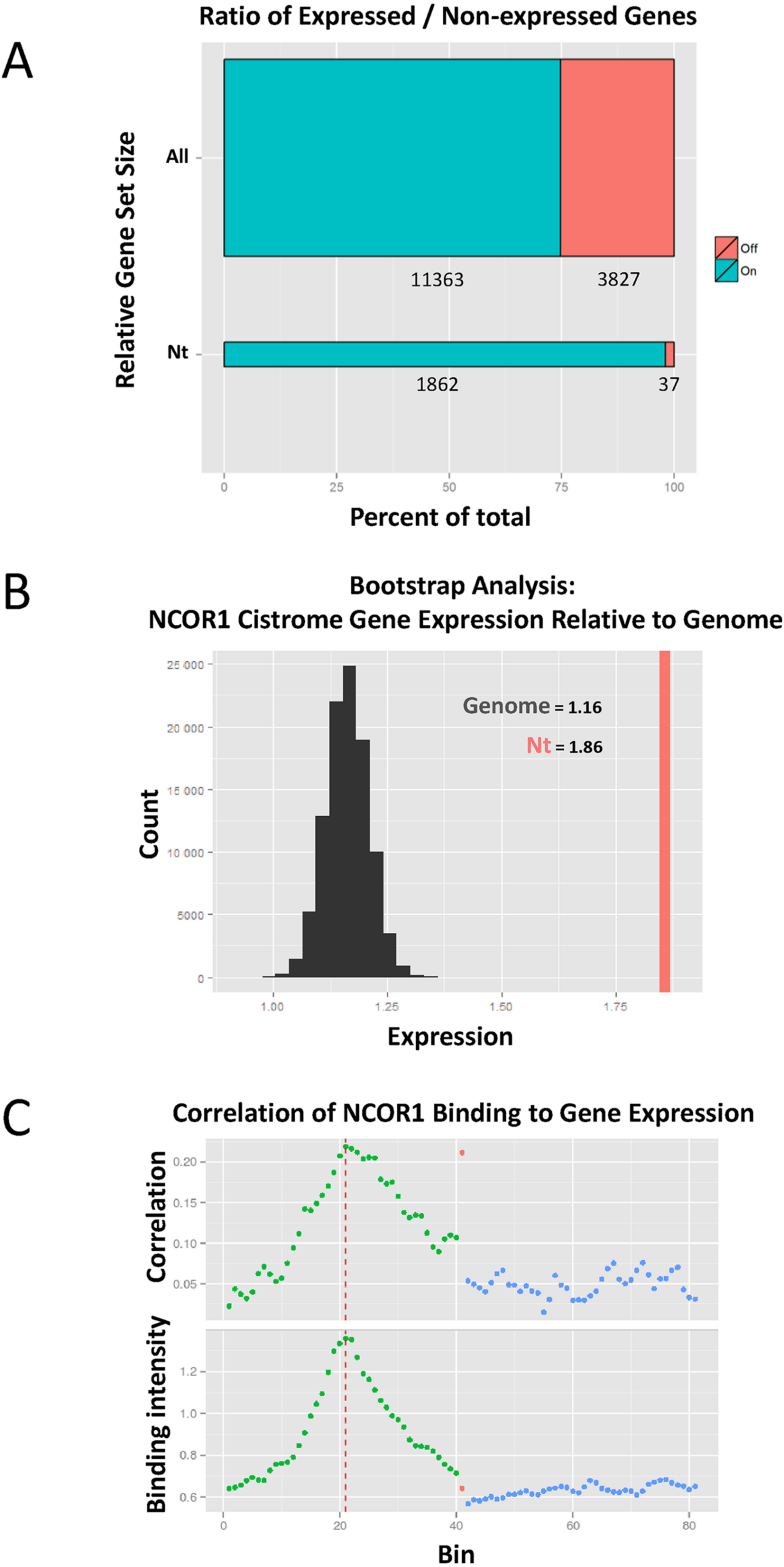
Annotating the NCOR1 cistrome relative to gene TSS and integration with RNA-Seq data in K562 cells. (**A**) ON/OFF proportions of RNA for all genes, and those associated with proximal NCOR1 binding. (**B**) Bootstrap analysis of NCOR1 genes versus the entire transcriptome. The red line on the right represents the Box-Cox transformed mean expression of the genes with NCOR1 binding with ±1 kb of TSS. The histogram represents the distribution of Box-Cox transformed mean transformed expression of randomly sampled (100 000 times) sets of the same size (1862 genes). NCOR1-associated genes are expressed at a significantly higher level than the background. (**C**) NCOR1 binding around genes is positively correlated with gene expression. Top: The correlation between expression and NCOR1 binding across the gene. Bottom: The average binding signal of NCOR1 is determined around the TSS and TTS each in 40 individual 100 bp bins (green and blue respectively) and the remaining average binding signal within the gene body. Vertical dotted line indicates TSS region while green, blue and red dots indicate values +/− 2 kb from the TSS, gene body and +/− 2 kb from TTS, respectively.

To investigate the relationships between proximal NCOR1 binding and gene expression, these ChIP-Seq data were related to normalized RNA-Seq data. In the first instance we examined genes with any significantly detectable level of expression (IDR value < 0.1), and therefore considered these as ‘on’ genes. By contrast ‘off’ genes were those that were reproducibly undetected in both samples (RPKM value: NA). In this manner, 11 363 genes (74.8%) were reliably detectable in the K562 RNA-seq data and 3827 genes (25.2%) were off. The relationship of the NCOR1 cistrome to the on and off gene sets was then examined. Eighteen hundred sixty-two of the 1899 genes (98.1%) that had proximal NCOR1 binding were on, associated with detectable expression, and only 37 genes (1.9%) that had NCOR1 proximal binding were undetectable (Figure 2A).

To test whether the detectable genes associated with proximal NCOR1 binding were altered in their expression compared to the background transcriptome, we simulated data for comparisons and bootstrap approaches were applied to test the significance of the association of NCOR1 binding and gene expression. Interestingly, this revealed that RNA expression for genes with a proximal NCOR1 binding peak had a mean normalized expression (RPKM) of 1.86, which was significantly higher (*P* < 1e^−5^) than would be expected by chance given the expression of the background transcriptome (mean RPKM of 1.16) (Figure 2B, Supplementary Table S2).

To establish confidence in this approach we also undertook the same analytical approaches with factors that are known to be either repressive of activating of gene expression Supplementary Figure S3. Specifically, we exploited ChIP-Seq data sets in K562 cells for the transcriptional repressor CTCF, and the gene repressive histone mark H3K27me3. In parallel we examined ChIP-Seq data from positive regulators of gene expression, namely c-MYC and activated RNA polymerase 2 (phosho-Pol-II). In each case the observed expression of genes associated with the proximal binding of the positive regulators of gene expression (c-MYC and phospho-Pol-II) was significantly higher than predicted by chance. By contrast, the observed expression of genes associated with the proximal binding of the negative regulators was associated with significantly lower gene expression.

We complemented these analyses by applying a ‘binning’ approach to map the average binding pattern of NCOR1 across genes associated with the NCOR1 cistrome (71). NCOR1-associated genes were divided into 81 bins each of a 100 bp that covered the TSS, gene body and TTS (Figure 2C). In the first instance, NCOR1 binding intensity was determined across genes and was found to be strongest at the TSS, with weak binding across the gene body and TTS. Interestingly, this binding pattern resembles that found for common activating histone marks such as H3K4me3 (data not shown). For each 100 bp bin, we then correlated NCOR1 binding intensity to gene expression across all genes. Interestingly, we found that NCOR1 binding positively correlated to gene expression in all bins, albeit modestly, with maximum correlation occurring at and around the TSS (Figure 2C). Lastly, these findings were supported by parallel analyses of ChIA-PET data for K562 cells, which revealed that the NCOR1 cistrome associates with inter- and intra-chromosomal interactions that are mediated by phospho-RNA-pol II occupancy (Supplementary Figure S4).

### Combining NCOR1 and other epigenomic data to define sub-cistromes

**Figure 3.**
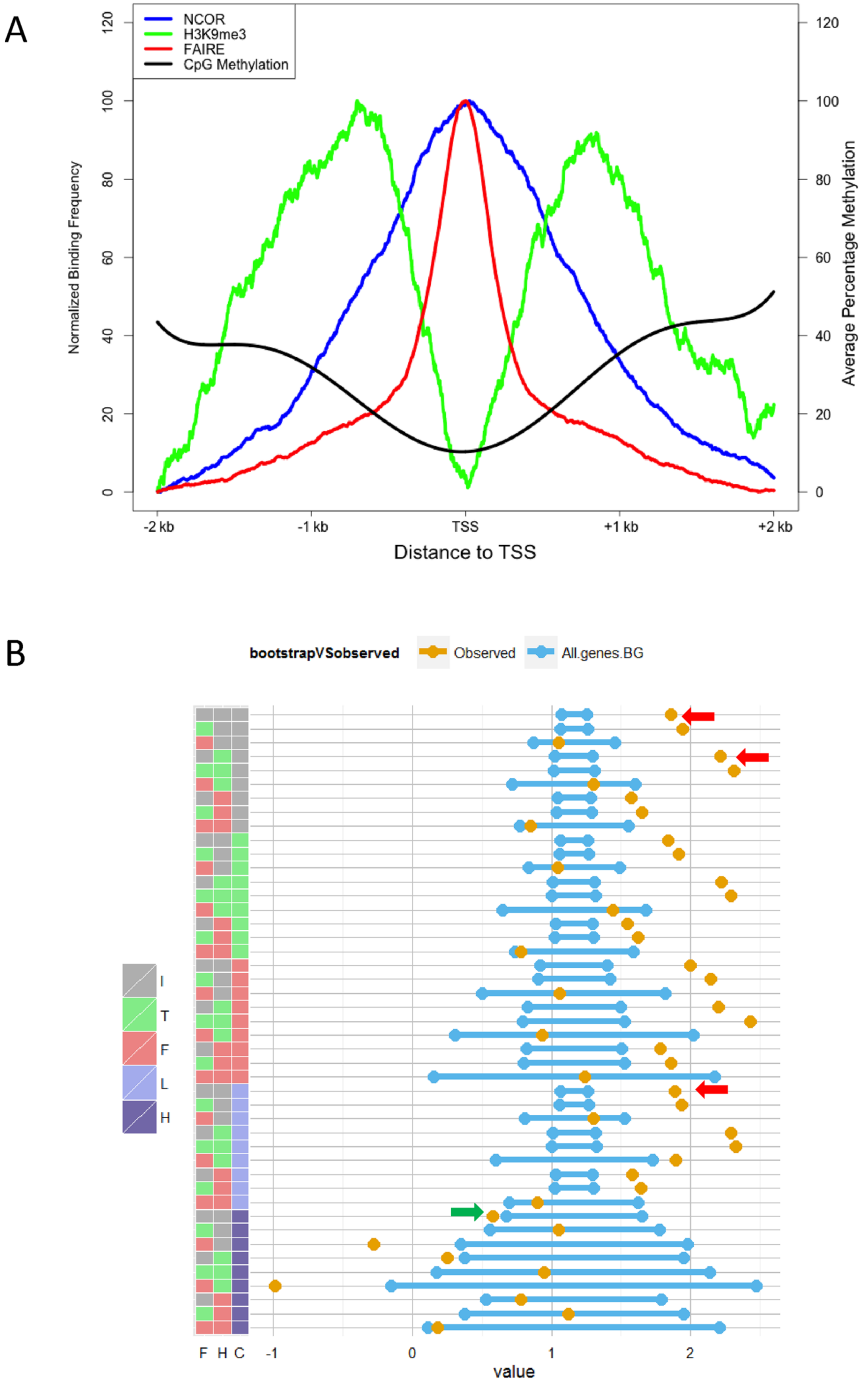
NCOR1 associates with highly expressed genes even when considering the effect of other chromatin modulating marks. (**A**) Profile of NCOR and H3K9me3 ChIP-Seq, FAIRE-seq and RRBS methylation data across TSS region. NCOR1 ChIP-Seq reads (blue line) tend to accumulate and peak around the TSS region with a spread of roughly 1 kb. This profile for FAIRE-seq data (red line) is similar to NCOR profile but much narrower, clustering on 500 bp around the TSS. H3K9me3 (green line) offers a bimodal profile, as expected by histone modifications, therefore peaking at +1 kb and −1 kb from the TSS. The average CpG methylation (black line) around the TSS is at the lowest at the TSS while it increases to ∼40–50% at +2 kb and −2 kb from the TSS regions. The x-axis indicates the distance from the TSS, the left y-axis indicates the normalized binding frequency for ChIP-Seq and FAIRE-seq data, while the right y-axis indicates the average percentage of methylation of the CpGs in the RRBS data set. (**B**) Expression analysis of NCOR1 subsets compared to background. The gold dot indicates the observed mean normalized expression, while the blue lines represent the 95% confidence intervals of the bootstrapped data for the same number of genes sampled from the entire protein coding transcriptome. Note that the width of the 95% confidence interval is dependent on the number of genes in the subset. The key to the left indicates the specific combinations of either FAIRE-Seq (F), H3K9me3 (H) or DNA CpG methylation (C). These three epigenomic states are considered as either independent (I), present (T) or absent (F) state. Furthermore when CpG was present it was considered in either a Low (L) or a High (H) state. Specifically, methylation status of each TSS region was calculated as the average methylation of all detectible CpGs within the region and were defined as low (C_l_, < 30%) or high (C_h_, > 30%).

We next considered the possibility that there were smaller subsets of significantly repressed genes contained within all the genes associated with the NCOR1 cistrome. To test this possibility we combined other epigenomic data sets to refine the cistrome-transcriptome analyses and examined the patterns of NCOR1 binding alongside FAIRE-status (open and closed chromatin), H3K9Me3 enrichment (a direct marker of NCOR1–HDAC3 activity) and DNA CpG methylation status (an indirect marker of NCOR1–HDAC3 and ZBTB33/KAISO activity). In the first instance we examined the binding intensity of these factors across the TSS of all genes (Figure 3A). This revealed that the distribution of NCOR1 binding more closely resembled that of the FAIRE-Seq data, and the position of open chromatin, but appeared to be somewhat reciprocal to the distribution of H3K9Me3 enrichment.

To test how the combination of factors related to gene expression, we defined all of the 1899 NCOR1-associated genes on the basis of either the presence (t), absence (f) or independence (i) of overlapping peaks for H3K9Me3 and FAIRE and the average CpG methylation (Low (l) High (h)). In this manner, we parsed the parental NCOR1 cistrome into 44 sub-cistromes and calculated the mean expression of the genes associated with each sub-cistrome, compared to the mean expression of randomly selected subsets of the same number of genes, using the bootstrap approach (Figure 3B, Supplementary Tables S2 and S3). In support of the initial finding for the whole NCOR1 cistrome, genes associated with 24 different sub-cistromes were also expressed at a significantly higher level than predicted by chance, compared to the simulated background (Supplementary Table S3).

Genes associated with NCOR1 peaks and either the presence or absence of H3K9Me3 peaks remained associated with significantly elevated gene expression. For example, N_t_F_i_H_t_C_i_ associated with 848 genes, 822 of which were detectable with a mean normalized expression of 2.21, and this was significantly elevated (*P* < 1e^−5^) compared to the randomly sampled background (Figure 3B and Supplementary Table S3). Similarly, the inclusion of FAIRE-seq peaks (N_t_F_t_H_i_C_i_) on the background of proximal NCOR1 peaks associated with 1461 genes, 1453 of which were detectable with a mean normalized expression of 1.91 that was significantly elevated (*P* < 1e^−5^). Finally, low levels of CpG methylation (N_t_F_i_H_i_C_l_) associated with 1531 detectable genes with a mean normalized expression of 1.88 that was also significantly elevated (*P* < 1e^−5^). Only the presence of high levels of CpG methylation (N_t_F_i_H_i_C_h_) was associated with repressed gene expression, of 64 genes with a mean normalized expression of 0.57 (*P* < 9.3e^−3^). Of the nine specific combinations containing high levels of CpG methylation, five were associated with gene repression. Of these, N_t_F_f_H_t_C_h_ resulted in the most significantly reduced expression (−0.98, *P* < 5.4e^−4^), however this subset was representative of only nine genes associated with the proximal NCOR1 cistrome.

We also considered an alternative manner to analyze the data by considering the choice of background from which to simulate data. Specifically, instead of sampling genes from the background of all protein coding genes we sampled from within the individual backgrounds to measure the impact of NCOR1 on gene expression. Applying the more parsimonious selection of background did not alter the finding that NCOR1-associated genes were more commonly elevated in expression than predicted by chance (Supplementary Figure S5). Taken together these findings support the approach simulating data using bootstrapping to define the impact of *cis*-binding factors on gene expression. Second, this approach also revealed that NCOR1 proximal binding is more commonly associated with significantly elevated gene expression than would be predicted by chance.

These results suggest that proximal NCOR1 binding in K562 cells is more commonly associated with significantly elevated expression of protein coding genes than would be predicted by chance. This remains generally true when extracting those genes where NCOR1 binding overlaps with FAIRE-Seq and H3K9me3 peaks. Only certain of the NCOR1 subsets when combined with high levels of CpG methylation were associated with genes that were expressed at a lower level than predicted by chance, although these represented sets of relatively small gene number.

### Biological processes and TF enrichment associated within the NCOR1 cistrome

To evaluate biological implications of genes associated with proximal NCOR1 binding, we used the DAVID Functional Annotation tool (v6.7). We examined enrichment of terms in all the genes associated with the proximal NCOR1 cistrome (N_t_) (*n* = 1899). The complete N_t_ transcriptome was significantly enriched (FDR *P* < 0.01) in nearly 80 keywords (e.g. phosphoprotein, acetylation and nucleus) and GO terms. Parallel analyses of these genes in GREAT also identified a range of biological processes, including erythrocyte differentiation.

Since the N_t_ gene set contains subsets of genes with substantially elevated (N_t_H_t_, N_t_C_l_) and repressed (N_t_C_h_) expression, we also extracted the unique genes in each of these subsets for further evaluation. Specifically, this subdivided the gene lists further (Supplementary Figure S2) and generated a series of unique gene lists for N_t_ (*n* = 134), N_t_H_t_ (*n* = 149), N_t_C_l_ (*n* = 874) and N_t_C_h_ (*n* = 43) (Supplementary Tables S2 and S3). By undertaking the same analyses with the unique gene lists, the identified terms became more focused. The unique N_t_ gene list was enriched for the Interpro terms, keywords and GO terms that centered on nucleosome function including the terms, acetylation, Histone core, nucleosome and chromatin organization. The N_t_H_t_ unique subset was enriched for two terms centered on proteasome function, although these did not survive FDR correction. The N_t_C_l_ subset recapitulated many aspects of the whole N_t_ list with acetylation being a prominent keyword. Finally, the N_t_C_h_ list was enriched in two terms related to control of NFκB signaling, although they did not survive FDR correction (Supplementary Table S4).

To identify TFs that were enriched within the proximal NCOR1 cistrome, we applied *de novo* motif search approaches. Mining the TSS ±1 kb region of the whole N_t_-associated transcriptome, in the first instance using Molecular Signatures Database (MSigDB) (79), revealed 12 different significantly enriched transcriptional motifs. The most enriched motif was for ETS domain-containing protein (ELK1) (2.00 hypergeometric fold enrichment) and ETS2, another ETS family member, was also significantly enriched. Similarly, MYC and MAZ motifs were both enriched. Interestingly, the only nuclear receptor motif enriched by this approach was Estrogen-related receptor alpha (ERRα).

Second, we used HOMER (72) to identify TF motifs that were specifically enriched in the NCOR1 peaks associated with the whole N_t_ transcriptome and the unique peaks associated with N_t_, N_t_H_t_, N_t_C_l_ and N_t_C_h_ transcriptomes. Analyses of these sub-cistromes identified a list of significantly enriched TF motifs (Table 1). These analyses also revealed that NCOR1 peaks in the whole N_t_ cistrome were significantly enriched for motifs associated with ETS family members including ETS, ELK1 as well as Friend leukemia integration 1 transcription factor (FLI1). Additionally, the sub-cistromes revealed enrichment of various motifs including GATA factors in N_t_; ER and MYB in N_t_H_t_; ETS homologous factor and zinc-finger proteins in N_t_C_l_; and ERRα and PPARγ in N_t_C_h_.

**Table 1.** Summary of the motifs identified by Homer

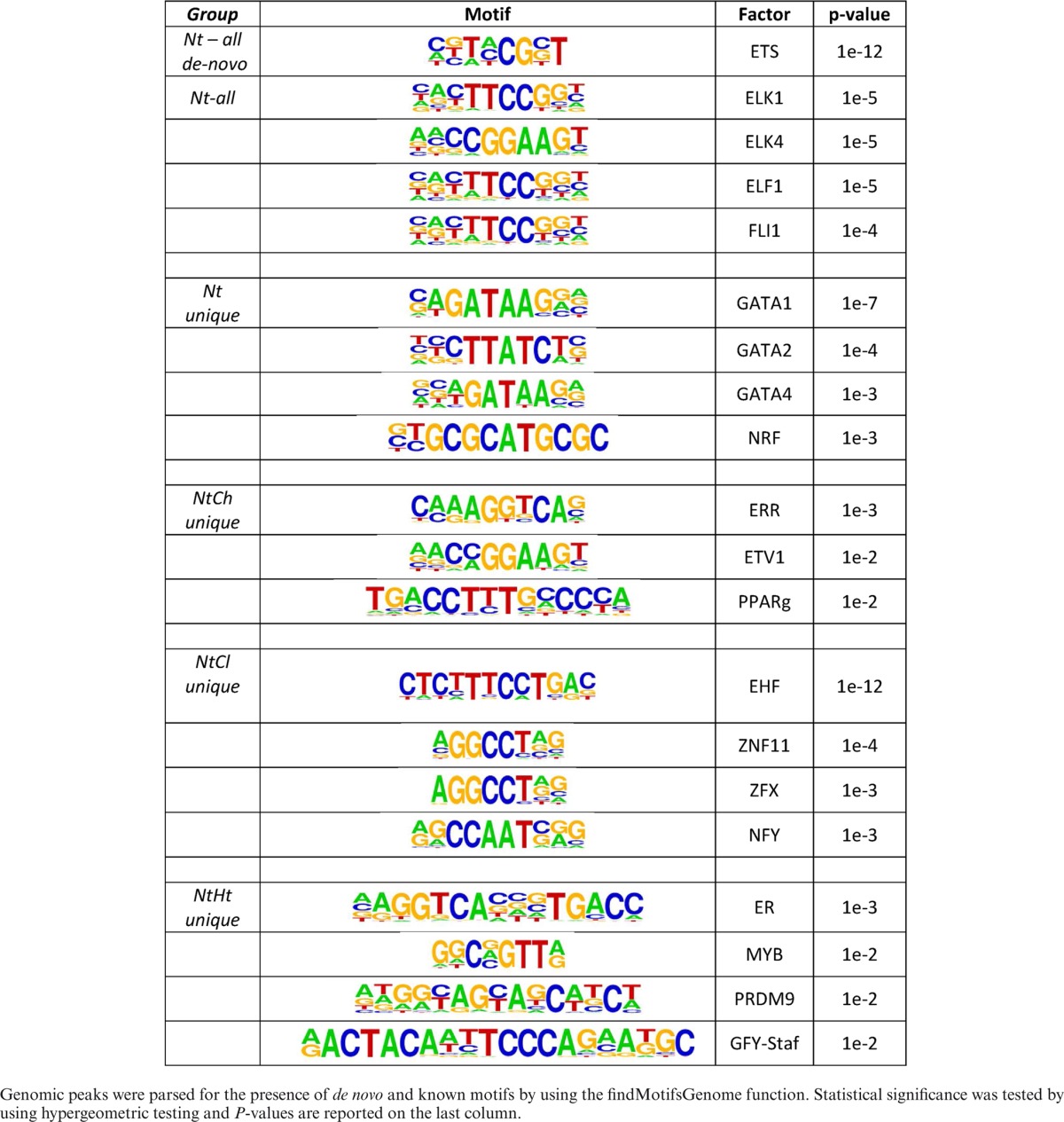

Genomic peaks were parsed for the presence of *de novo* and known motifs by using the findMotifsGenome function. Statistical significance was tested by using hypergeometric testing and *P*-values are reported on the last column.

**Figure 4.**
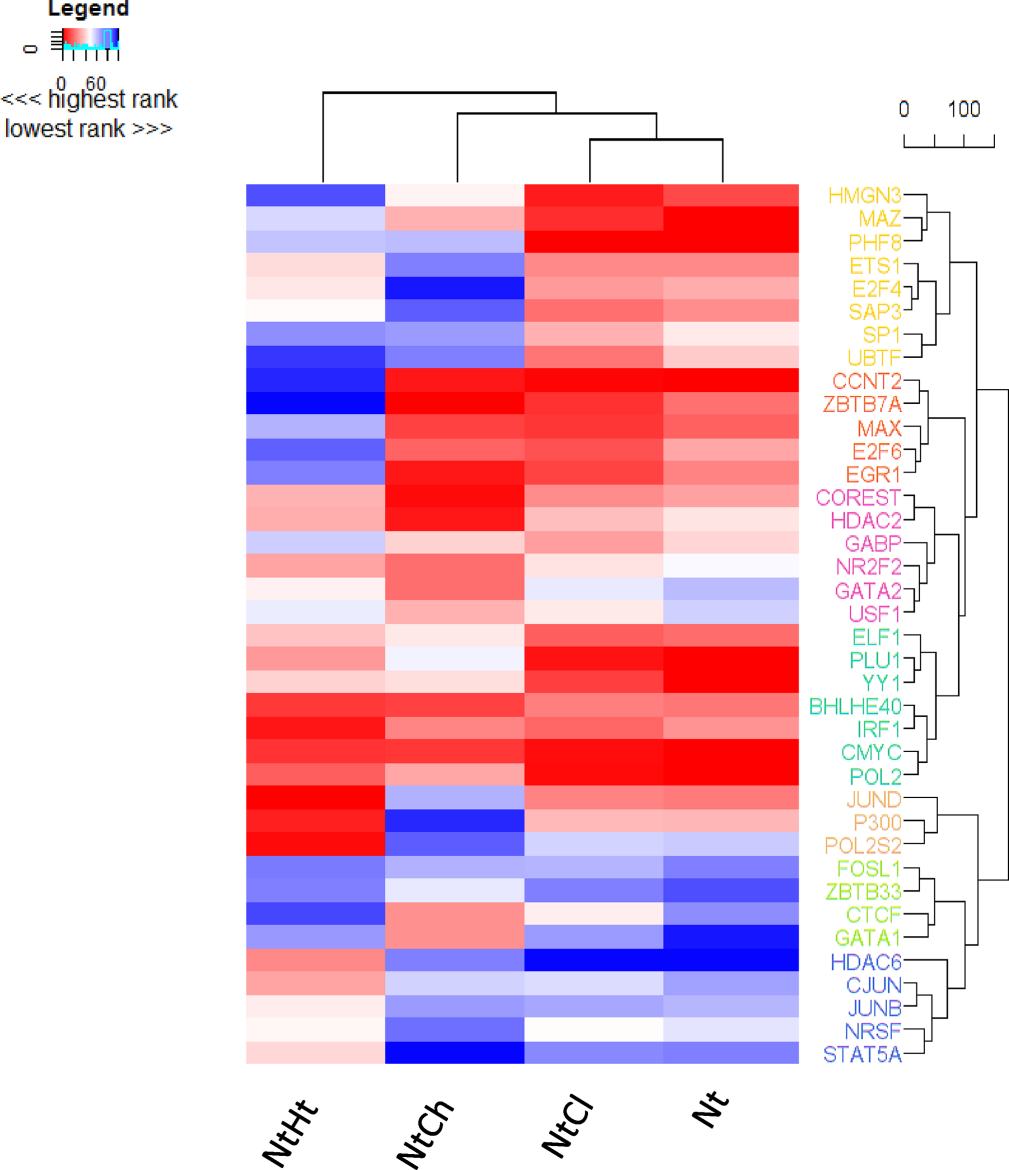
Ranking of TFs enriched in genes associated with NCOR1 binding. The indicated gene sets were investigated for enrichment of TF binding using the ENCODE ChIP-Seq Significance Tool. The ranked data are presented as a heatmap to indicate how the rank order of TF enrichment differs between the gene sets. Red indicates increased ranking and blue decreased ranking.

Finally, we took advantage of the repository of ENCODE TF ChIP-Seq data in K562 cells using the ENCODE ChIP-Seq Significance Tool, using the TSS ±5 kb region of the whole N_t_-associated transcriptome and the unique gene sets associated with N_t_, N_t_H_t_, N_t_C_l_ and N_t_C_h_. Overall, comparing the ranking in percentile of the common TFs across the unique gene lists can be summarized in seven unique clusters (Figure 4) representing the differential binding occurring across the four conditions. These analyses revealed significant biological binding of a number of the same TF motifs that were predicted to be enriched. Again, ETS family members and MYC family members (MAZ and MAX) were highly ranked in the unique N_t_ and N_t_C_l_ cistromes. In the presence of H3K9me3 (N_t_H_t_) a cluster of TFs, including HDAC6, JUN members, NRSF and STAT5A had elevated ranking, indicated by being in red (Figure 4). The N_t_C_h_ genes, which were significantly repressed, were associated with a relatively loss of a cluster (in blue) including ETS1 and accumulation of CTCF and GATA1, as well as RCOR1/CoREST and HDAC2.

### NCOR1 knockdown leads to down-regulation of genes associated with the proximal NCOR1 cistrome

To examine the functional relationship between NCOR1 binding and gene expression, we generated K562 cells with a stable knockdown of *NCOR1* (K562-shNCOR1 (Supplementary Figure S6A). In the first instance, we examined cell viability and found that K562-shNCOR1 cells grew significantly slower after 72 h than the control cells (Supplementary Figure S6B). Subsequently, we examined the impact of NCOR1 knockdown on gene expression. A standard microarray analytical workflow was used to establish significantly detectable genes in the K562- K562-shCTR and K562-shNCOR1 cells, as well as the significant DEG. In the first instance, a significant correlation was revealed between the basal gene expression in the K562 cells as measured by RNA-seq by the ENCODE consortium and the K562-shCTR cells in the current study (*r* = 0.603, *P* < 2.2e^−16^) (Supplementary Figure S6C). Furthermore, genes associated with proximal NCOR1 binding and for which there were probes on the Illumina microarray were expressed at a significantly elevated level in K562-shCTR cells compared to the background genome (Supplementary Figure S6D), supporting the initial analyses (Figure 2).

**Figure 5.**
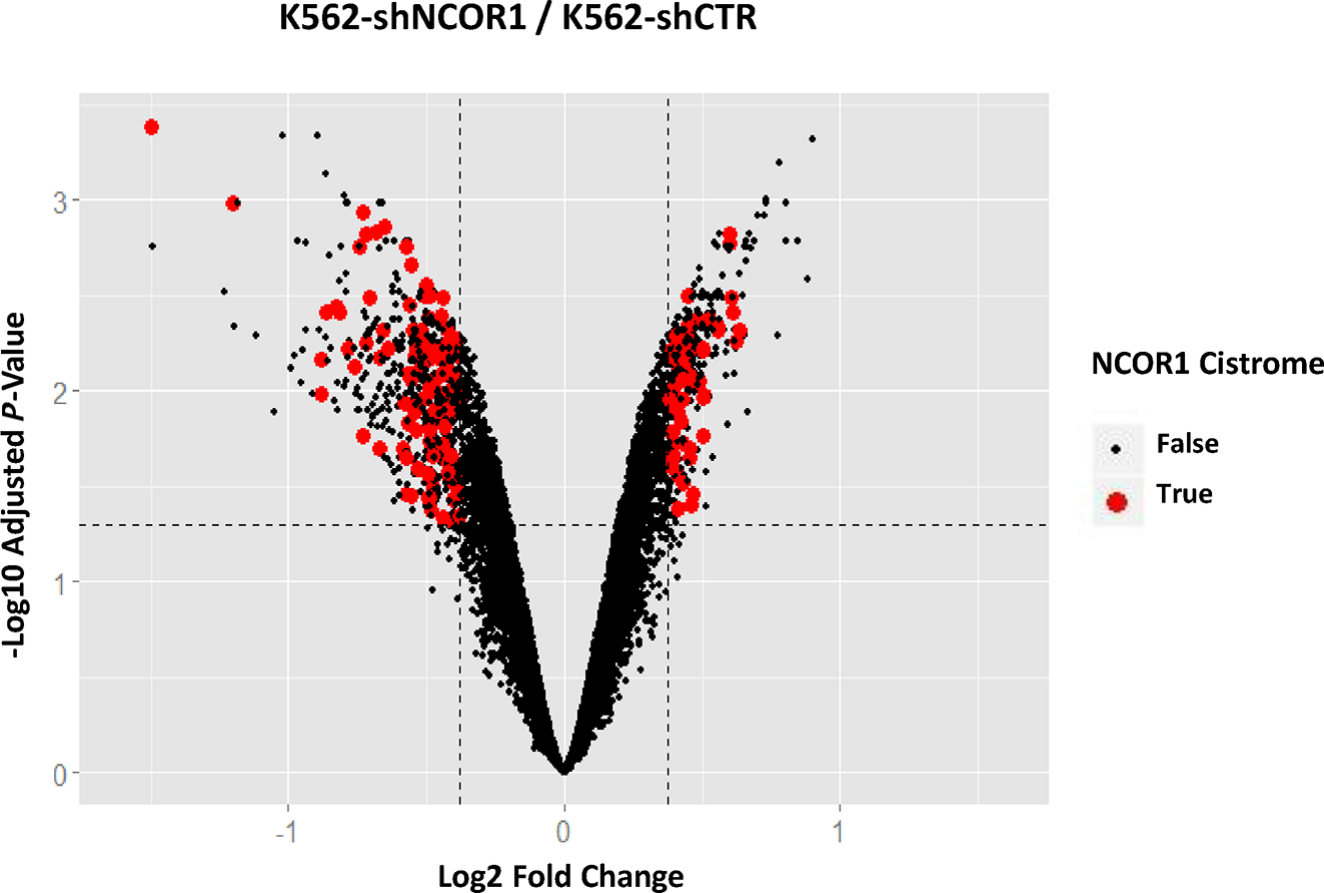
Expression analysis in K562-shNCOR1 cells. Volcano plot showing DEGs in K562 shNCOR1 cells compared to K562 shControl cells. Red dots show the genes associated with proximal NCOR1 binding (±1 kb of TSS). Dotted lines show the fold change and *P*-value cut offs. Genes down-regulated upon NCOR1 knockdown were significantly enriched for the genes associated with the proximal NCOR1 cistrome.

The DEGs following NCOR1 knockdown revealed that a greater proportion of genes were down-regulated (*n* = 404, average fold change = 1.45) than up-regulated (*n* = 283, average fold change = 1.38) (Figure 5, Supplementary Table S5). Subsequently, we compared how these up- and down-regulated genes relate to the NCOR1 cistrome-associated genes identified above. Comparing the DEGs following knockdown of NCOR1 to the NCOR1 cistrome-associated genes revealed that genes down-regulated were significantly enriched within the NCOR1 cistrome (hypergeometric test, *P* = 0.0044), whereas genes with elevated expression upon NCOR1 knockdown were not enriched (Figure 5, Supplementary Table S5). Interestingly, down-regulated NCOR1 cistrome-associated genes in K562-shNCOR1 cells (*n* = 98) were among the most highly expressed NCOR1 cistrome genes in K562-shCTR cells (Supplementary Figure S7A–C). For instance, the mean expression of these 98 genes was 9.40 in K562-shCTR cells and was significantly elevated over the background transcriptome (8.617) as well as when compared to all genes within NCOR1 cistrome (8.875). However, in K562-shNCOR1 cells, the mean expression of these 98 genes was 8.980, only slightly elevated over the background transcriptome (8.618) and not significantly different from the expression of genes within the NCOR1 cistrome. This observation suggests that the NCOR1 plays a critical role in maintaining expression patterns of a subset of genes with substantially elevated transcription in K562 cells.

We also considered how NCOR1 knockdown selectively impacted genes associated with the sub-cistromes associated with altered expression (Figures 3 and 4). The volcano plots in Supplementary Figure S8 reveal that the DEGs following NCOR1 knockdown were singicnatly enriched in the repressed genes associated the whole NCOR1-dependent transcriptome (N_t_) (Supplementary Figure S8A) and the overlap with low levels of CpG methylation (N_t_C_l_) (Supplementary Figure S8C).

These findings, combined with the observed positive association of NCOR1 binding with elevated gene expression *in silico*, suggest that proximal binding of NCOR1 in K562 cells has a functional role in maintaining significantly elevated gene expression, and its loss results in dampened expression of highly expressed genes.

To evaluate the function and transcriptional complexes involved in the regulation of the DEGs, we again queried the DEGs with DAVID and ENCODE ChIP-Seq Significance Tool (Supplementary Table S4, Supplementary Figure S9). Among the 404 down-regulated genes following NCOR1 knockdown, 98 overlapped with the NCOR1 cistrome genes and among these, acetylation was a significantly enriched GO term, reflecting the N_t_ and N_t_C_l_ sub-cistromes. No enrichment terms survived multiple testing correction in the up-regulated genes following NCOR1 knockdown. A number of TF clusters were more enriched in the down-regulated than in the up-regulated gene list and includes ATF3, CJUN and CEBPB. The up-regulated genes were highly enriched in the interferon gamma receptor (IRF1), STAT5A and TRIM28.

### Mining the NCI-60 data to identify the druggability of genes associated with proximal NCOR1 binding

To investigate how genes associated with proximal NCOR1 binding may be selectively drugged, we leveraged findings from the NCI-60 data set which compiles gene expression profiles and drug sensitivity profiles across 60 cancer cell lines. We built an analytical workflow founded on the CellMiner tool (73). For each drug sensitivity profile (*n* = 20 509) we correlated gene expression profiles (*n* = 26 062) across the 60 cell lines. In this manner we generated drug sensitivity associated gene sets for all compounds available in the NCI-60 data set. Finally, we examined all drug-associated gene sets for NCOR1 cistrome enrichment (hypergeometric tests, FDR = 0.05) to identify drugs that target genes associated with proximal NCOR1 binding.

For simplicity, we focused our analyses on FDA approved drugs in the matrix (*n* = 108). These analyses revealed a number of drugs with sensitivity associated gene sets (both positive and negative associations) that were significantly enriched within the proximal NCOR1 cistrome-associated genes (N_t_) (Table 2). Interestingly, gene sets associated with the BCR-ABL inhibitors Nilotinib and Imatinib were the two most significantly enriched within the proximal NCOR1 cistrome-associated genes (Supplementary Figure S10). For example, Imatinib sensitivity positively correlated with the relative expression of 351 genes across the NCI-60 cell lines, 74 of which were found within the NCOR1 cistrome, representing a 21% overlap (adj. *P*-value 3.86 e^−17^). Similarly, several drugs had negatively associated gene sets enriched in the NCOR1 cistrome including the tyrosine kinase inhibitors (TKIs) Dasatinib and Erlotinib.

**Table 2.** NCI-60 drugs with sensitivity associated gene sets significantly enriched within the NCOR1 cistrome

Drug name	Mechanism of action	Indications	NCI-60: Correlated genes (positive)	NCOR1: Cist. genes	Overlap (n)	Overlap (%)	Adj. *P*-value
Nilotinib	TKI	CML	531	1753	109	20.53	6.19E-24
Imatinib mesylate	TKI	CML	351	1753	74	21.08	3.86E-17
Lomustine	Alkylating agent	various cancers	1299	1753	154	11.86	5.38E-11
Carmustine	Alkylating agent	various cancers	1984	1753	205	10.33	3.51E-09
Vemurafenib	BRAF inhibitor	melanoma	4059	1753	368	9.07	3.95E-09
Thioguanine	DNA synthesis inhibitor	leukemia, CML	435	1753	66	15.17	6.42E-09
Methotrexate	Antifolate	various cancers, autoimmune diseases	718	1753	81	11.28	2.99E-05
Arsenic Trioxide	Apoptosis inducer	leukemia	362	1753	48	13.26	4.58E-05
Pipamperone	Antipsychotic	schizophrenia	326	1753	44	13.5	6.80E-05
Thiopurine (6MP)	DNA synthesis inhibitor	leukemia	328	1753	44	13.41	7.87E-05
Vorinostat	HDACi	CTCL, various cancers	164	1753	27	16.46	1.09E-04
Oxaliplatin	Alkylating agent	colorectal cancer	578	1753	65	11.25	2.53E-04
Tamoxifen citrate	Hormone	breast cancer	1731	1753	156	9.01	6.77E-04
Dacarbazine	Alkylating agent	various cancers	798	1753	81	10.15	9.77E-04
Paclitaxel	Taxane	various cancers	131	1753	21	16.03	1.19E-03
Raloxifene hydrochloride	Hormone	breast cancer, osteoporosis	869	1753	86	9.9	1.41E-03
Dromostanolone Propionate	Hormone	cholesterol maintenance	386	1753	43	11.14	4.98E-03
Dasatinib	TKI	CML	1878	1753	179	9.53	6.68E-05
Erlotinib hydrochloride	TKI	various cancers	764	1753	86	11.26	1.16E-04
Irofulven	Alkylating agent	various cancers	1162	1753	119	10.24	1.59E-04
Hydroxyurea	DNA synthesis inhibitor	leukemia, antiretroviral	555	1753	66	11.89	2.65E-04
Nelarabine	DNA synthesis inhibitor	ALL	412	1753	51	12.38	8.16E-04
Pipobroman	Alkylating agent	various cancers	261	1753	35	13.41	2.48E-03
Dexrazoxane	Topoisomerase 2 inhibitor	Cardioprotection	699	1753	73	10.44	3.67E-03
Melphalan	Alkylating agent	various cancers	371	1753	43	11.59	8.42E-03

Summary of enrichment analyses comparing FDA approved drug sensitivity associated gene sets (positive, above; negative, below) with NCOR1 cistrome genes. All drugs with significant (FDR < 0.01) NCOR1 cistrome enrichment are shown.

**Figure 6.**
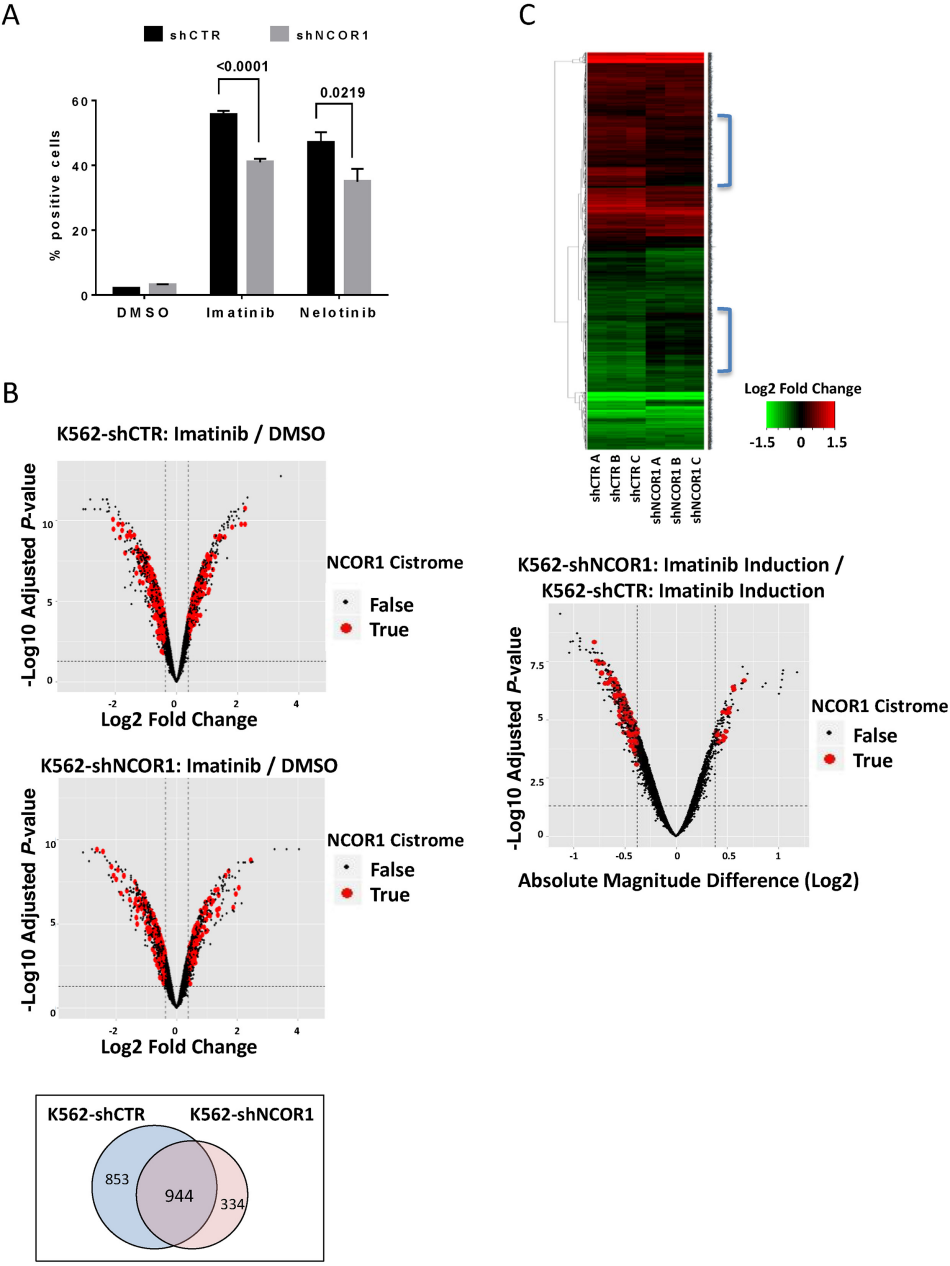
Identification of drugs with sensitivity associated gene sets enriched with proximal NCOR1 binding using NCI-60 data. **(A)** Erythroid differentiation in response to Imatinib and Nilotinib. Cell were treated with 500 nM Imatinib or 24 nM Nilotinib for 72 h. Subsequently, cells were stained with benzidine solution. The images were analyzed with *CellProfiler* to determine the proportion of benzidine-positive cells and K562-shNCOR1 cells were compared to K562-shCTR using t-test. K562-shNCOR1 cells showed significantly reduced erythroid differentiation upon treatment for the both the drugs tested. **(B)** Volcano plots depicting expression changes upon Imatinib treatment (500 nM, 48 h) in K562-shCTR cells (upper) and K562-shNCOR1 cells (middle). Genes with proximal NCOR1 binding (Nt) are shown in red for DEGs (FDR = 0.05, FC > 1.3). Venn diagram showing comparison of DEGs induced by Imatinib treatment in K562-shCTR and K562-shNCOR1 cells (bottom). **(C)** Heatmap of Imatinib induced Log2 Fold Change corresponding to all DEGs in both K562-shCTR and K562-NCOR1 cells (*n* = 2131). Biological replicates are shown, and both sample and genes are clustered using Euclidean distance and complete linkage. Also, the magnitude of absolute expression change induced by Imatinib in each cell line was directly compared.

To test the predictions made concerning the druggability of the NCOR1 cistrome, we examined the impact of NCOR1 knockdown on Imatinib and Nilotinib induced cell responses and gene expression. Cells were treated with either 500 nM Imatinib or 24 nM Nilotinib and cell growth and differentiation was examined (80–82). NCOR1 knockdown did not significantly impact the cell growth inhibition effect of either Imatinib or Nilotinib (data not shown), but it did significantly alter the capacity of Imatinib and Nilotinib to induce erythroid differentiation as measured by the benzidine assay. K562-shNCOR1 cells were significantly less sensitive to the induction of differentiation in response to either Imatinib or Nilotinib (Figure 6A). These results suggest that the genes associated with the proximal NCOR1 cistrome regulate erythroid differentiation in response to Imatinib and Nilotinib.

To assess the NCOR1-dependent Imatinib responsiveness at the transcriptional level, we undertook microarray analyses following Imatinib treatment (500 nM, 48 h) in K562-shCTR and K562-shNCOR1 cells. Both DEG lists were significantly enriched within the NCOR1 cistrome (Figure 6B, Supplementary Table S5). For instance, 22.7% of DEGs upon Imatinib treatment in K562-shCTR cells had proximal NCOR1 binding and similarly 23.1% of DEGs upon Imatinib treatment in K562-shNCOR1 cells had proximal NCOR binding. We therefore next directly compared DEGs in K562-shCTR and K562-shNCOR1 cells. First, Imatinib induced more DEG in K562-shCTR cells (1797) than in K562-shNCOR1 cells (1278). Of note, while there remained a large overlap of DEGs in the isogenic cell pair (944), there were a substantial number of unique DEGs in each cell line (Figure 6C). Also, in comparing the absolute magnitude of transcriptional changes among DEGs in each cell line, we found that transcriptional effects were largely dampened in magnitude in K562-shNCOR1 cells, and there was a skewing to the left, with more genes lost in expression in the Imatinib treated K562-shNCOR1 cells compared to Imatinib treated control cells (Figure 6C—bottom panel). Overall, these findings reveal that NCOR1 binding in K562 cells is enriched near the Imatinib modulated transcriptome, and that NCOR1 plays an important role in mediating the transcriptional effects induced upon Imatinib treatment in BCR-ABL positive cells.

The number of the genes in each list is not unique, since other lists of DEGs share them. In order to identify subset of genes either uniquely regulated by or independent from Imatinib and NCOR1, we intersected all the DEG lists obtained from all the binary combinations of the microarray conditions (Supplementary Figure S11). Expression of 2153 genes (column 8) was dependent upon Imatinib but independent from NCOR1 status since they were differentially expressed in both K562-shNCOR Imatinib treated cells and K562-shCTR Imatinib treated cells. Conversely, six genes (column 9) were dependent upon NCOR1 status but not Imatinib stimulation since they were differentially regulated in NCOR1 knockdown cells and in K562-shNCOR1 Imatinib treated versus K562-shControl Imatinib treated cells. One hundred eighteen genes (columns 11 and 15) were instead commonly regulated by Imatinib and NCOR1, since they are differentially expressed upon Imatinib treatment in any condition and differentially regulated by NCOR1 knockdown, suggesting that the BCR-ABL lesion uniquely regulates a subset of the NCOR1 cistrome. Lastly, 44 genes were commonly differentially expressed in all the conditions (column 16).

To place these findings in a broader context we compared the enrichment of these DEGs with publically available K562 microarray data upon Imatinib treatment (GSE1922) and Nilotinib treatment (GSE19567). The NCOR1-dependent DEGs were significantly enriched in these microarray data with 22.9% and 20.8% of DEGs associated the NCOR1 cistrome being DEGs in the drug treated DEG lists (Supplementary Table S6). Also, integrating DEGs in CD34+ blasts from CML patients treated with Imatinib (400 mg daily, 1 week) were also similarly enriched for proximal NCOR1 binding (22.2%) (83). However, genes associated with Imatinib therapy resistance in patients at 12 months (GSE14671) (76) were not significantly enriched in the genes associated with proximal NCOR1 binding. These enrichment analyses suggested that NCOR1 binding plays an important role in regulating expression of genes that are involved in the transcriptional responses induced by Imatinib treatment.

## DISCUSSION

NCOR1 imparts important roles to control gene expression through interactions with a wide array of different TFs. However, to date the relationships between NCOR1 (or NCOR2/SMRT) binding and gene expression have not been significantly investigated on the genome-wide scale in human cells and therefore the global function of these large and complex proteins remains enigmatic. Therefore, the goal of the current study was to fill this knowledge gap and generate a more complete biological view of NCOR1 function by establishing statistically robust associations across the genome, epigenome and transcriptome, which in turn combine to impact cell phenotypes. Specifically, we hypothesized that the biological function of NCOR1 can be revealed by *in silico* integration of multiple omic data sets followed by *in vitro* testing of functional consequences (Figure 1).

To meet this knowledge gap we took advantage of NCOR1 ChIP-Seq data generated in the ENCODE Tier 1 cell line, K562. The fact that K562 cells are a Tier 1 cell line is critical as this means that a prodigious volume of data are available with a remarkable potential for combinatorial and integrative analyses. Other workers, including the NCI-60 consortium, have also extensively investigated K562 cells. We related NCOR1 binding at proximal promoter regions of protein coding genes to open chromatin and the levels of both H3K9me3 and DNA CpG methylation, and in turn to gene expression. Although there are established roles for NCOR1 to act as a corepressor at candidate loci, these genomic approaches found that NCOR1 binding across the genome was more frequent around detectable than undetectable genes, compared to the background protein coding genome, and was more associated with significantly elevated gene expression than predicted by chance.

We considered several explanations for why these significant relationships should be revealed on the genomic scale. One reason for these potentially surprising findings is that the genome-wide functions of NCOR1 and NCOR2/SMRT, and indeed many other co-regulatory proteins, are often inferred from candidate gene loci. There is a large, and rapidly growing, volume of studies that exploit ChIP-Seq approaches to define cistrome patterns. However, within these studies it is less common to see the application of statistical approaches to define cistrome-transcriptome relationships. Often these relationships are inferred by co-incident knockdown or knockout approaches combined with gene expression analyses. The sets of genes can then be established where the factor of choice binds and is modulated by intervention and candidate loci are given as illustrations of genome-wide phenomena. Whilst this is a powerful approach, it has some limitations and the current study complements these approaches and provides a framework for relating cistrome and transcriptome data by exploiting bootstrapping approaches to simulate data for comparison. To the best of our knowledge this is the first time this approach has been applied to relate genome-wide protein binding to genome-wide gene expression.

Aside from technical or statistical explanations, we postulated from these observations that either 1. NCOR1 is recruited to highly expressed genes to limit their expression and allow for their rapid down-regulation in response to cell stimuli; or 2. NCOR1 when bound at proximal regions, actually functions as a transcriptional co-activator.

To test these possibilities we stably knocked down expression of NCOR1 in K562 cells and undertook microarray analyses. This revealed that a greater number of genes were down-regulated than up-regulated, and that the down-regulated genes were most significantly enriched within the genes that were most significantly elevated associated with NCOR1 cistrome and shared enrichment for the same keywords (e.g. acetylation). Our *in silico* analyses focused on NCOR1 proximal binding function, and these *in vitro* findings suggest that the transcriptional co-activator function is predominant at the genome-wide level as the number of up-regulated genes was smaller. Collectively, these findings favor the hypothesis that NCOR1 when bound at or close to the TSS of genes acts most commonly as a coactivator. These data are supported by a literature of findings concerning coactivator function of NCOR2/SMRT, which can act as a coactivator for p53 (40) and ERα (41).

A third possible explanation is that NCOR1 has a dual function acting as a spatially dependent coactivator and corepressor, therefore inhibiting or promoting transcription based on the genomic landscape it is recruited to. A relevant example of this modulatory function is the Lysine Specific Demethylase 1A (KDM1A/LSD1) that was recently described as corepressor and coactivator for the androgen receptor (AR) in prostate cancer (84). In the current study, the dynamic NCOR1 cistrome, modulating its transcriptional targets, may reflect the changing distribution between distal or proximal gene relationships, presumably being modulated in response to environmental stimuli or disease status. The apparent role of the proximal Nt cistrome targets to regulate Imatinib sensitivity suggests a role for oncogenic BCR-ABL to influence NCOR1 association with these genes.

It is therefore important to understand the TF interactions of NCOR1. To achieve this we exploited *de novo* prediction approaches (e.g. HOMER) and the significant volume of ChIP-Seq data from ENCODE. In this manner, we mined TF associations with proximal NCOR1 binding to identify which were most significant. These analyses of the whole NCOR1 cistrome both revealed common enrichment of a number of TF families principally including ETS family members. ETS1 family members have been identified previously to interact with NCOR1 (85,86) and another identified family member FLI1, which is known to participate in the control of erythorid differentiation (87,88). Similarly ELF1 was identified in both approaches but has not been reported before for NCOR1. Analyses of the unique subsets also revealed significant interactions of TFs, for example GATA interactions appeared to enrich within the unique genes associated N_t_, and has not previously been reported. MYC and MYC family members also were enriched, and an interaction with MYC has also been suggested (89). In trastuzumab-treated breast cancer cells NCOR2/SMRT interacts with MYC (90).

By contrast to these relatively understudied interactions, there is a very large literature on the association between NCOR1 and ERα (91–93) and PPARs (14,94–96). However, it is worth noting that ERα motifs were identified in the N_t_H_t_ subset of genes, the interaction with PPARγ was only identified for the N_t_C_h_ subset; thus PPARγ associations were only identified in 43 out of the 1899 NCOR1-associated genes, or 2% of the targeted transcriptome. Another NCOR1 interaction identified was with ZBTB33/KAISO, and this interaction is enriched mostly in the N_t_C_h_ subset of significantly repressed genes. This is an established NCOR1 interacting protein initially identified for being recruited by the NCOR1 complex to repress gene expression (33,97). Later results also associated ZBTB33/KAISO with a role in active transcription (98) suggesting a potential dual role for the NCOR1-ZBTB33/KAISO complex as locus specific transcriptional activator/repressors.

These findings were given greater potential clinical significance as we developed an approach based on the NCI-60 to identify the relationships between multi-gene signatures and drug sensitivity. Using this approach, we identified that genes associated with sensitivity to the BCR-ABL inhibitors Imatinib and Nilotinib were significantly enriched in the NCOR1 proximal cistrome. These findings also point to a role for BCR-ABL oncogenic actions to support NCOR1 interactions at the gene sites identified.

In the first instance we examined the changes in gene expression following NCOR1 knockdown and revealed that the knockdown of NCOR1 modulated sensitivity to Imatinib induced differentiation in K562 cells, and these gene expression patterns were significantly enriched in both the Imatinib-dependent gene signature of K562 cells and in CML cells from patients who were sensitive to Imatinib. Furthermore ETS family members were enriched in the genes where NCOR1 appeared to be functioning as a coactivator.

Of note, of the 25 drugs listed in Table 2, four are specifically indicated for CML (Imatinib, Nilotinib, Dasatinib and Thioguanine), with nine targeted to leukemia and lymphoma in general, and at least 22 are commonly utilized in the treatments of various malignancies. Of these, several provided as intriguing candidates for further study. For instance, Vorinostat effectively antagonizes the NCOR1–HDAC complex by blocking histone deacetylase activity, which is thought to lead to widespread increased histone acetylation and open chromatin. However, recent findings have revealed that critical anticancer actions provided by Vorinostat are also linked to gene repressive events (99) suggesting that antagonizing NCOR1 function has more complex effects on transcription than previously attributed. Also, several hormone related drugs were identified in our analysis including the selective estrogen receptor modulators Tamoxifen and Raloxifene and the synthetic androgen Drostanolone. This is interesting due to the well-established interactions between NCOR1 and nuclear receptors including the estrogen and ARs. However, the BCR-ABL inhibitors Nilotinib and Imatinib were substantially enriched in our analyses relative to other FDA approved drugs.

Imatinib revolutionized the treatment of CML in patients harboring the BCR-ABL translocation, which is present in K562 cells. While Imatinib remains highly effective in these patients, resistance mechanisms do often develop. We therefore chose to examine the effect of NCOR1 loss on Imatinib sensitivity. In terms of the understanding of Imatinib sensitivity mechanisms, it is clear that NCOR1 plays a role in erythrocyte differentiation (100) and that there are links between NCOR1 and CMYC to regulate Imatinib sensitivity (101,102). Similarly, other workers have established synergy between the HDACi Vorinostat and Imatinib (103,104). Again, this raises the complex question of what is Vorinostat's role in this process and whether it predominantly inhibits gene expression within the NCOR1 cistrome or rather enhances it. These data suggest that NCOR1 is linked to the malignant cistrome associated with the BCR-ABL genetic lesion.

## CONCLUSIONS

Overall, we have described a novel approach to define how *cis*-binding factors impact the cistrome, transcriptome and phenotype relationships and exploited NCOR1 to illustrate this approach. These approaches identified that in BCR-ABL positive K562 cells, NCOR1 when bound in proximal positions commonly functioned as a positive regulator of gene expression and that these genes overlapped with a druggable hub centered on Imatinib sensitivity genes. By focusing on NCOR1 these analyses are given further relevance as this protein has emerged as a major disease driver in a range of cancers (24,25) and is implicated in metabolic syndromes (105,106). Thus the findings generated have potential for translation across tumor types and to other disease states.

## Supplementary Material

SUPPLEMENTARY DATA
