# Supplementary material for: Integrative genomic analysis in K562 chronic myelogenous leukemia cells reveals that proximal NCOR1 binding positively regulates genes that govern erythroid differentiation and Imatinib sensitivity
Source: Nucleic Acids Res. 2015 Jun 27;43(15):7330–48. doi: 10.1093/nar/gkv642 (PMC4551916; doi:10.1093/nar/gkv642)
Supplement: SUPPLEMENTARY DATA [file supp_43_15_7330__index.html]

Integrative genomic analysis in K562 chronic myelogenous leukemia cells reveals that proximal NCOR1 binding positively regulates genes that govern erythroid differentiation and Imatinib sensitivity — SUPPLEMENTARY DATA 

# Integrative genomic analysis in K562 chronic myelogenous leukemia cells reveals that proximal NCOR1 binding positively regulates genes that govern erythroid differentiation and Imatinib sensitivity

## SUPPLEMENTARY DATA

- SUPPLEMENTARY DATA
- SUPPLEMENTARY DATA
