# Supplementary material for: Integrative genomic analysis in K562 chronic myelogenous leukemia cells reveals that proximal NCOR1 binding positively regulates genes that govern erythroid differentiation and Imatinib sensitivity
Source: Nucleic Acids Res. 2015 Jun 27;43(15):7330–48. doi: 10.1093/nar/gkv642 (PMC4551916; doi:10.1093/nar/gkv642)
Supplement: SUPPLEMENTARY DATA [file supp_gkv642_nar-00838-x-2015-File003.pptx]

## Slide 1
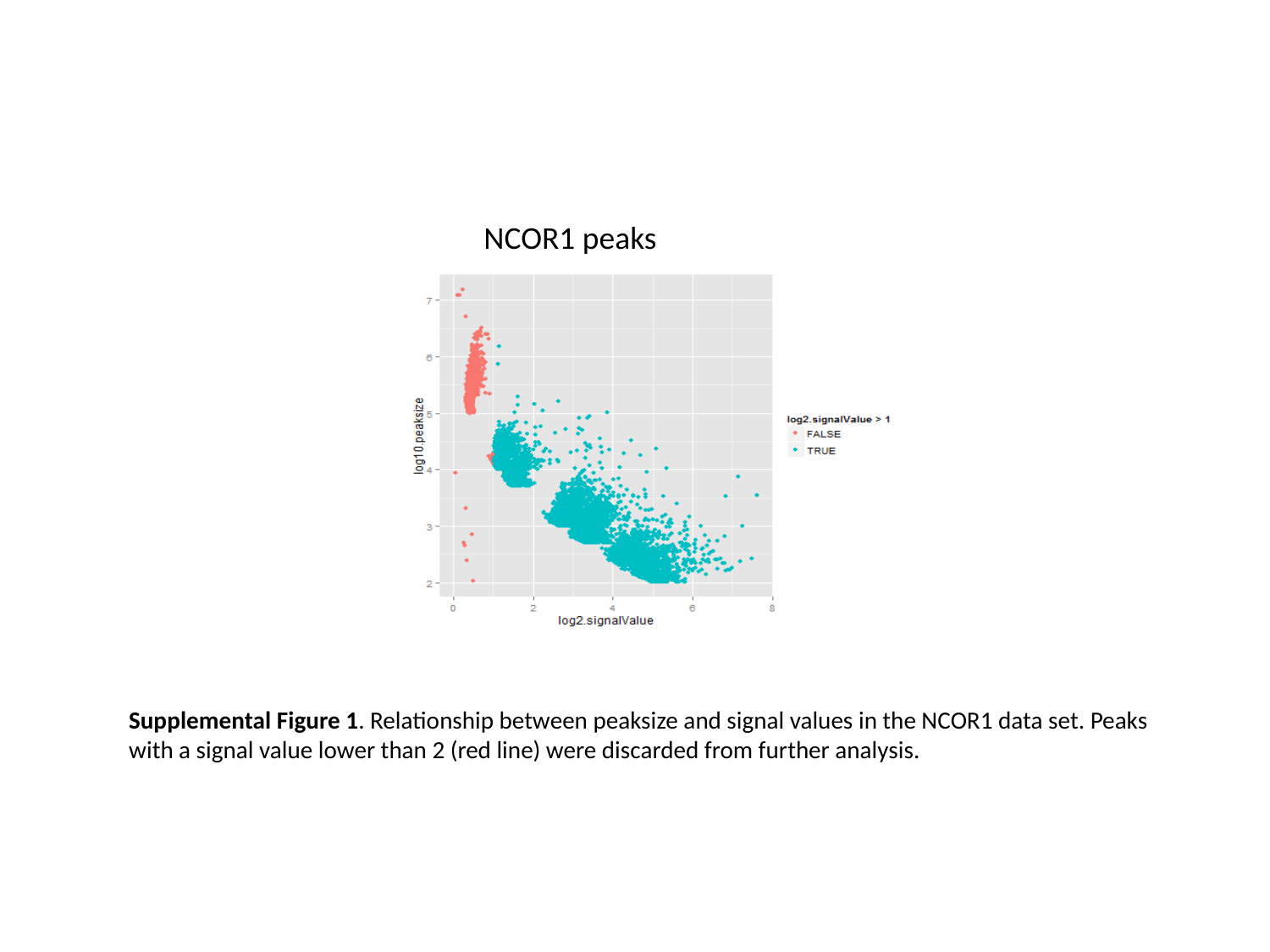

NCOR1 peaks
Supplemental Figure 1. Relationship between peaksize and signal values in the NCOR1 data set. Peaks with a signal value lower than 2 (red line) were discarded from further analysis.

## Slide 2
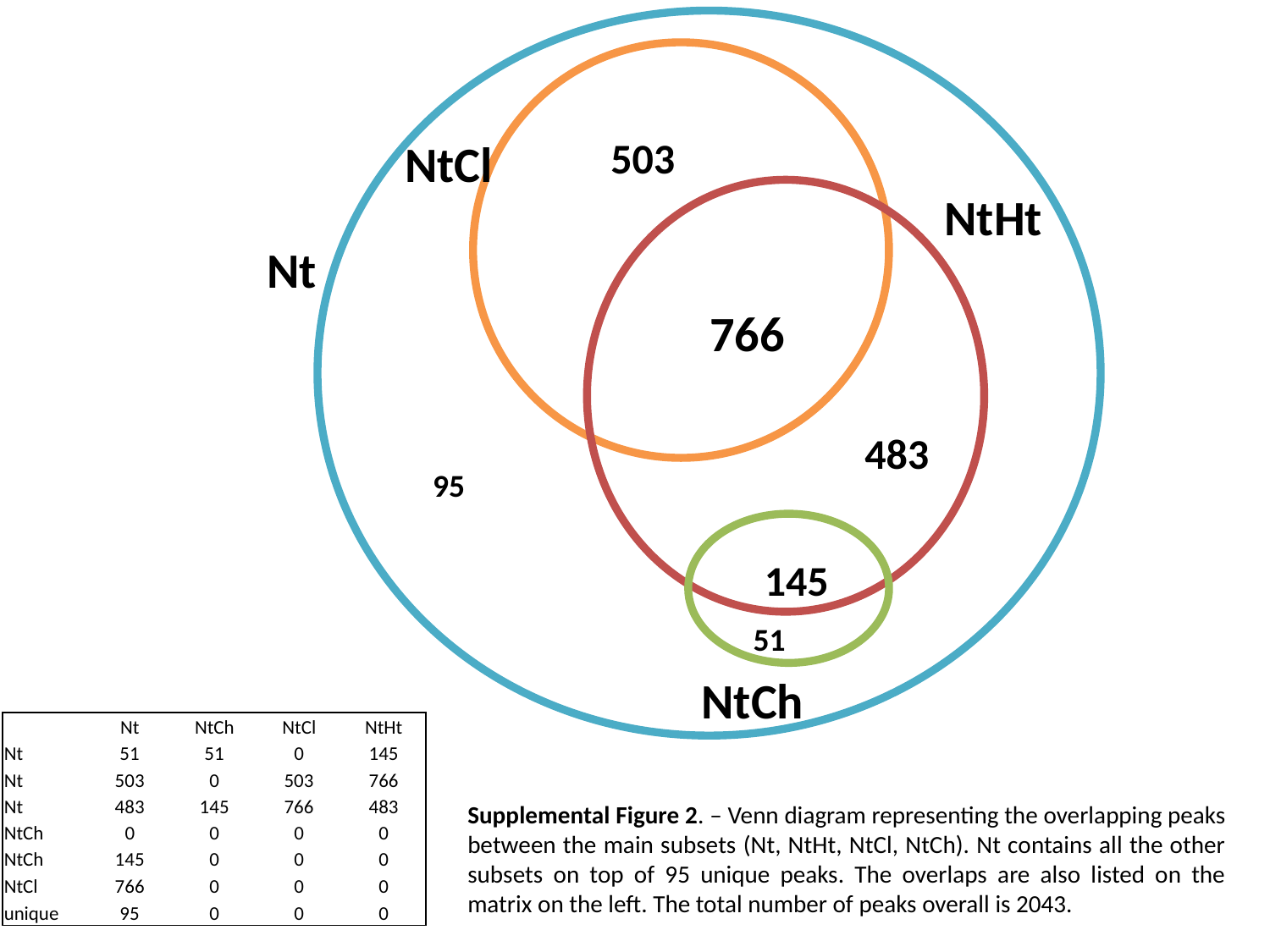

503
NtCl
NtHt
Nt
766
483
95
145
51
NtCh
| | Nt | NtCh | NtCl | NtHt |
| --- | --- | --- | --- | --- |
| Nt | 51 | 51 | 0 | 145 |
| Nt | 503 | 0 | 503 | 766 |
| Nt | 483 | 145 | 766 | 483 |
| NtCh | 0 | 0 | 0 | 0 |
| NtCh | 145 | 0 | 0 | 0 |
| NtCl | 766 | 0 | 0 | 0 |
| unique | 95 | 0 | 0 | 0 |
Supplemental Figure 2. – Venn diagram representing the overlapping peaks between the main subsets (Nt, NtHt, NtCl, NtCh). Nt contains all the other subsets on top of 95 unique peaks. The overlaps are also listed on the matrix on the left. The total number of peaks overall is 2043.

## Slide 3
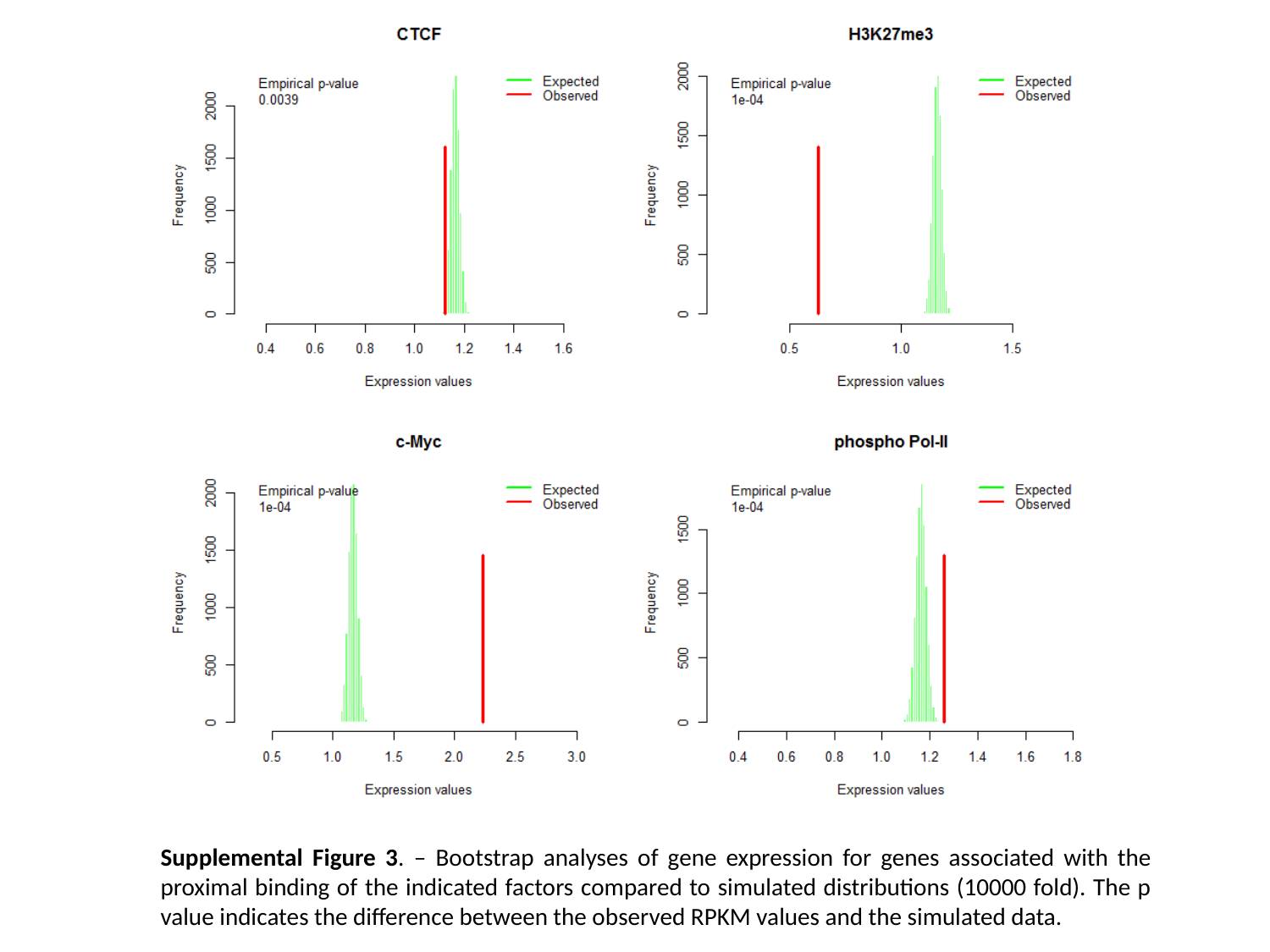

Supplemental Figure 3. – Bootstrap analyses of gene expression for genes associated with the proximal binding of the indicated factors compared to simulated distributions (10000 fold). The p value indicates the difference between the observed RPKM values and the simulated data.

## Slide 4
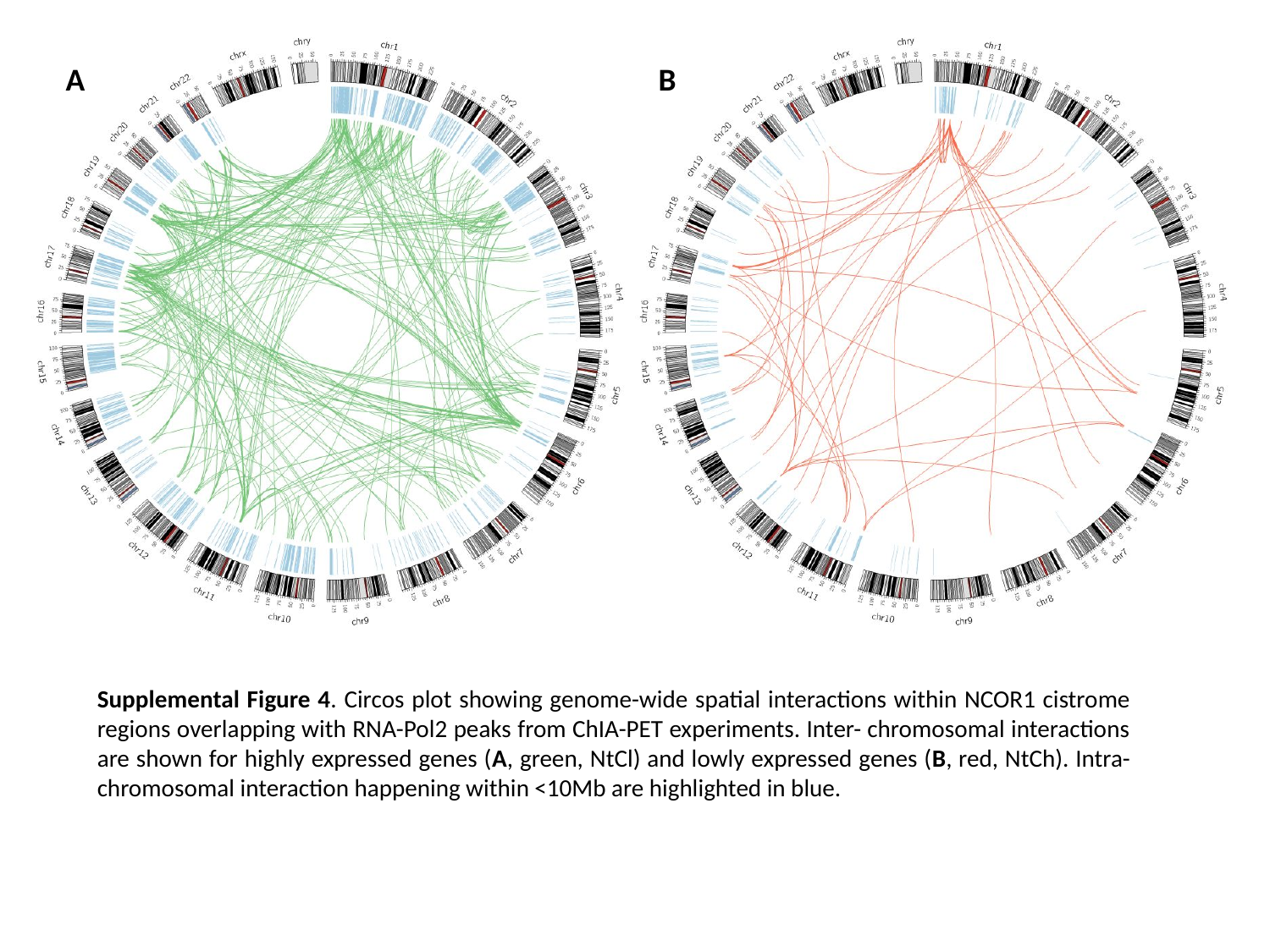

A
B
Supplemental Figure 4. Circos plot showing genome-wide spatial interactions within NCOR1 cistrome regions overlapping with RNA-Pol2 peaks from ChIA-PET experiments. Inter- chromosomal interactions are shown for highly expressed genes (A, green, NtCl) and lowly expressed genes (B, red, NtCh). Intra-chromosomal interaction happening within <10Mb are highlighted in blue.

## Slide 5
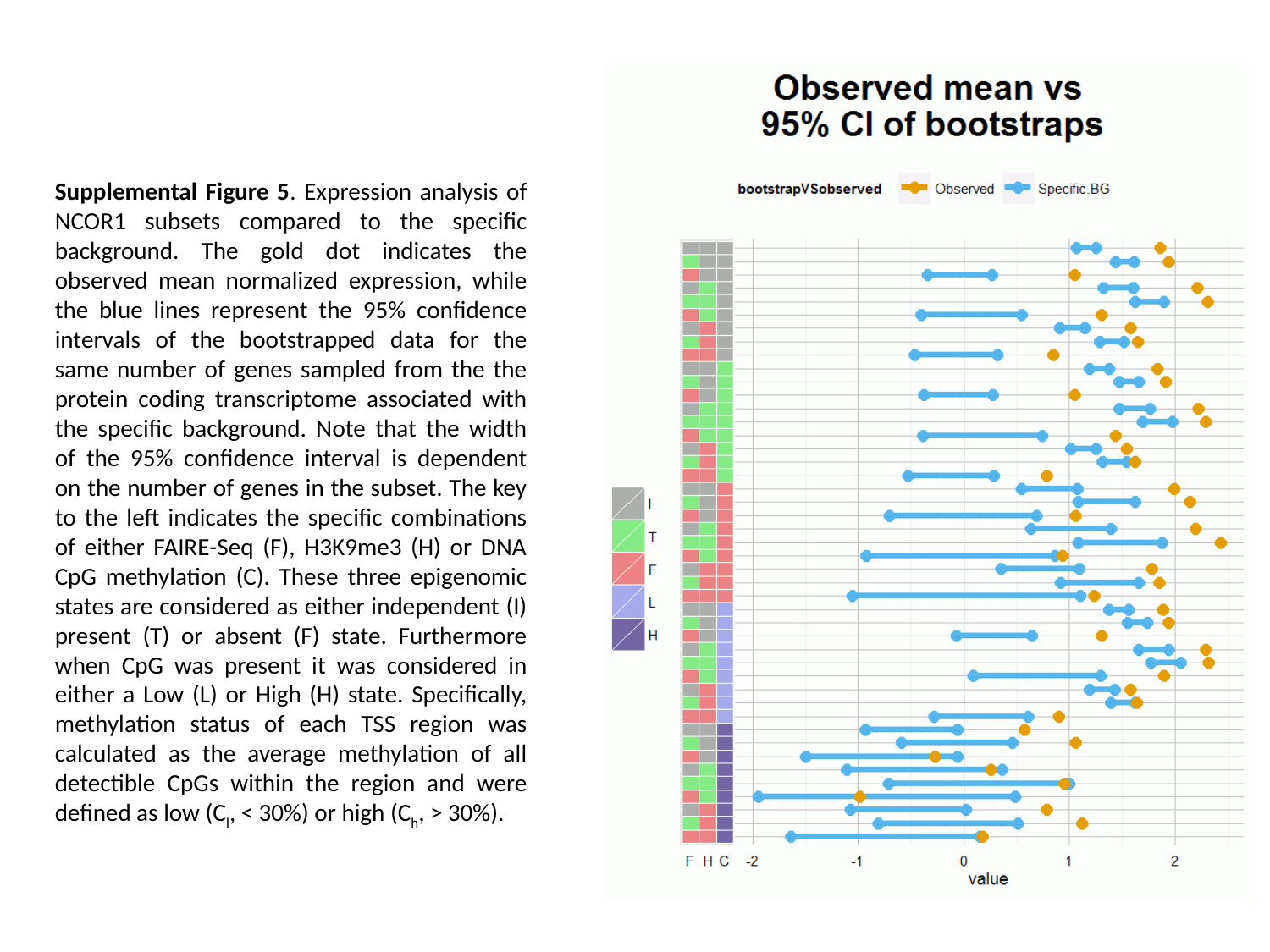

Supplemental Figure 5. Expression analysis of NCOR1 subsets compared to the specific background. The gold dot indicates the observed mean normalized expression, while the blue lines represent the 95% confidence intervals of the bootstrapped data for the same number of genes sampled from the the protein coding transcriptome associated with the specific background. Note that the width of the 95% confidence interval is dependent on the number of genes in the subset. The key to the left indicates the specific combinations of either FAIRE-Seq (F), H3K9me3 (H) or DNA CpG methylation (C). These three epigenomic states are considered as either independent (I) present (T) or absent (F) state. Furthermore when CpG was present it was considered in either a Low (L) or High (H) state. Specifically, methylation status of each TSS region was calculated as the average methylation of all detectible CpGs within the region and were defined as low (Cl, < 30%) or high (Ch, > 30%).

## Slide 6
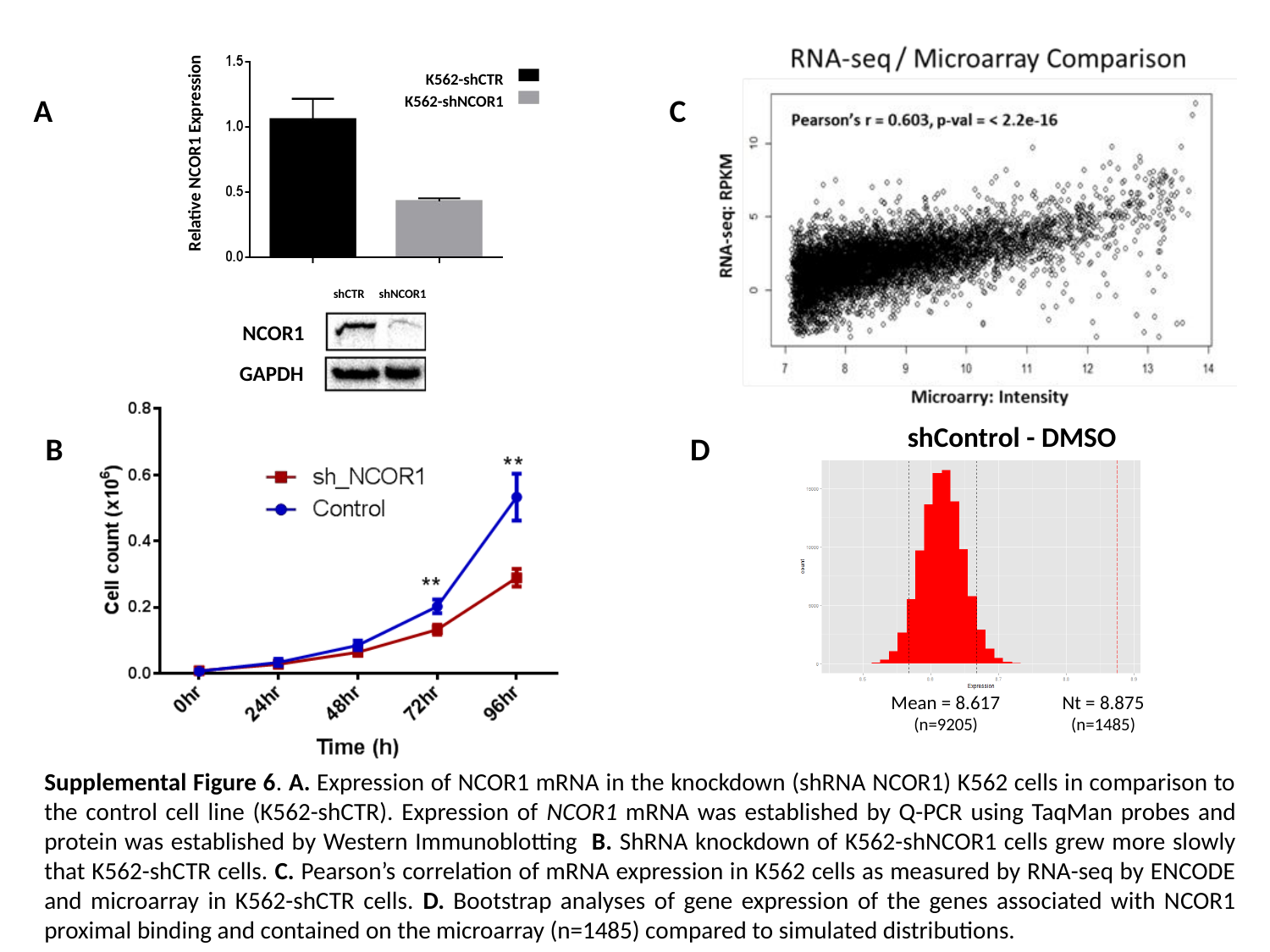

K562-shCTR
A
K562-shNCOR1
C
Relative NCOR1 Expression
shCTR
shNCOR1
NCOR1
GAPDH
B
A
shControl - DMSO
Mean = 8.617
(n=9205)
Nt = 8.875
 (n=1485)
D
Supplemental Figure 6. A. Expression of NCOR1 mRNA in the knockdown (shRNA NCOR1) K562 cells in comparison to the control cell line (K562-shCTR). Expression of NCOR1 mRNA was established by Q-PCR using TaqMan probes and protein was established by Western Immunoblotting B. ShRNA knockdown of K562-shNCOR1 cells grew more slowly that K562-shCTR cells. C. Pearson’s correlation of mRNA expression in K562 cells as measured by RNA-seq by ENCODE and microarray in K562-shCTR cells. D. Bootstrap analyses of gene expression of the genes associated with NCOR1 proximal binding and contained on the microarray (n=1485) compared to simulated distributions.

## Slide 7
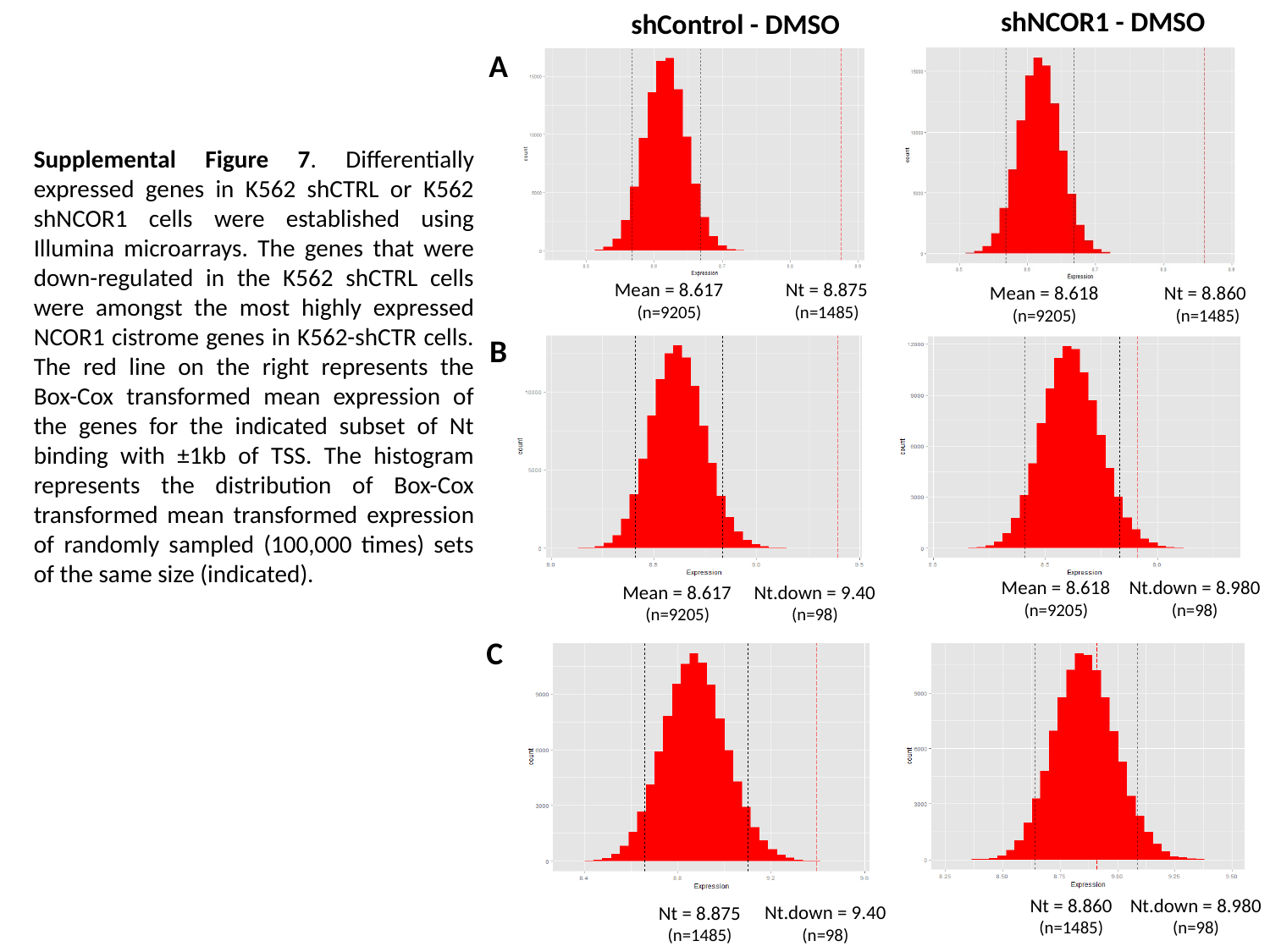

shNCOR1 - DMSO
Mean = 8.618
(n=9205)
Nt = 8.860
(n=1485)
shControl - DMSO
Mean = 8.617
(n=9205)
Nt = 8.875
 (n=1485)
A
Supplemental Figure 7. Differentially expressed genes in K562 shCTRL or K562 shNCOR1 cells were established using Illumina microarrays. The genes that were down-regulated in the K562 shCTRL cells were amongst the most highly expressed NCOR1 cistrome genes in K562-shCTR cells. The red line on the right represents the Box-Cox transformed mean expression of the genes for the indicated subset of Nt binding with ±1kb of TSS. The histogram represents the distribution of Box-Cox transformed mean transformed expression of randomly sampled (100,000 times) sets of the same size (indicated).
B
Mean = 8.618
(n=9205)
Nt.down = 8.980
 (n=98)
Nt.down = 9.40
 (n=98)
Mean = 8.617
(n=9205)
C
Nt.down = 8.980
 (n=98)
Nt = 8.860
 (n=1485)
Nt.down = 9.40
 (n=98)
Nt = 8.875
 (n=1485)

## Slide 8
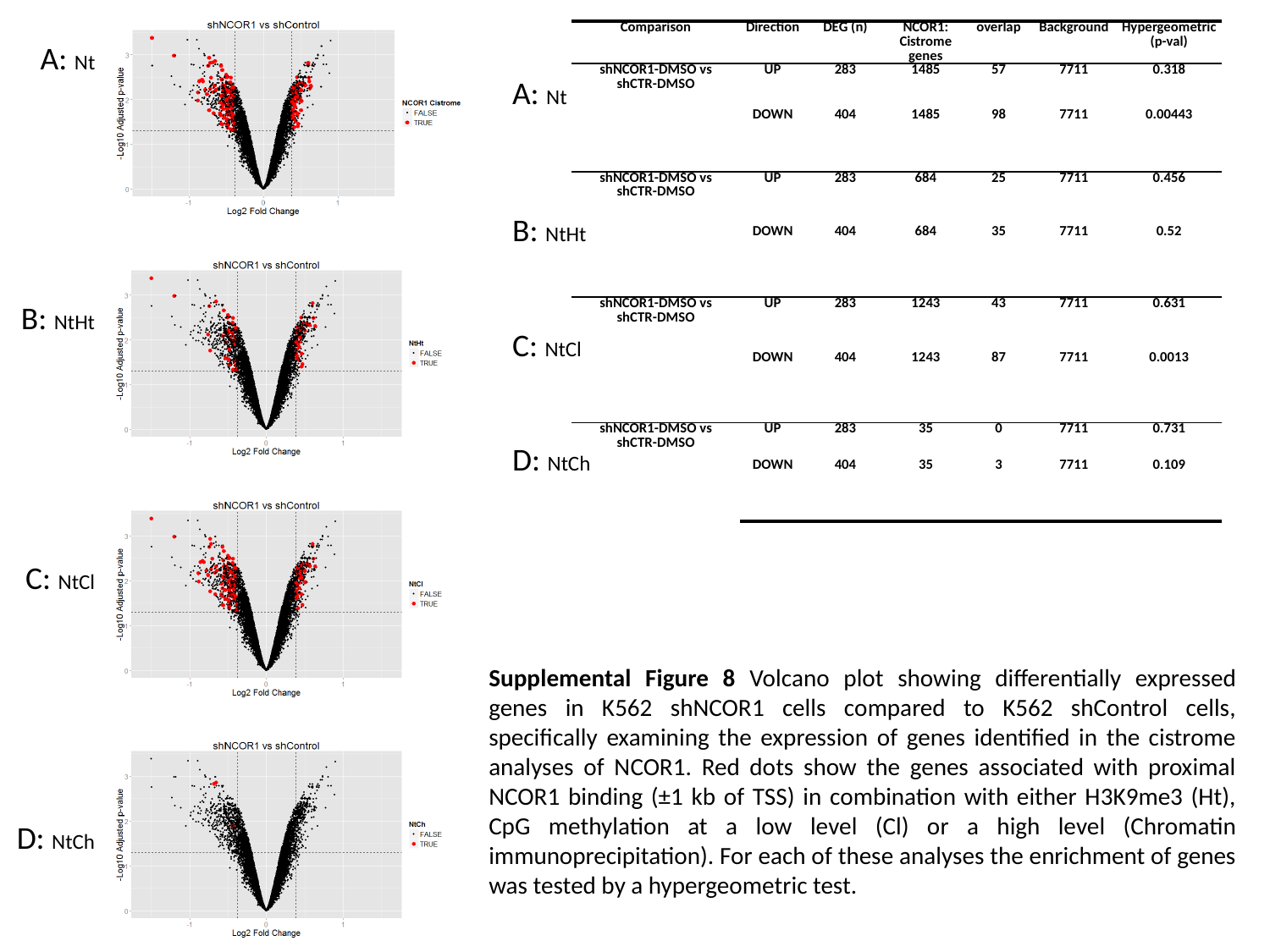

| Comparison | Direction | DEG (n) | NCOR1: Cistrome genes | overlap | Background | Hypergeometric (p-val) |
| --- | --- | --- | --- | --- | --- | --- |
| shNCOR1-DMSO vs shCTR-DMSO | UP | 283 | 1485 | 57 | 7711 | 0.318 |
| | DOWN | 404 | 1485 | 98 | 7711 | 0.00443 |
| shNCOR1-DMSO vs shCTR-DMSO | UP | 283 | 684 | 25 | 7711 | 0.456 |
| | DOWN | 404 | 684 | 35 | 7711 | 0.52 |
| shNCOR1-DMSO vs shCTR-DMSO | UP | 283 | 1243 | 43 | 7711 | 0.631 |
| | DOWN | 404 | 1243 | 87 | 7711 | 0.0013 |
| shNCOR1-DMSO vs shCTR-DMSO | UP | 283 | 35 | 0 | 7711 | 0.731 |
| | DOWN | 404 | 35 | 3 | 7711 | 0.109 |
A: Nt
A: Nt
B: NtHt
B: NtHt
C: NtCl
D: NtCh
C: NtCl
Supplemental Figure 8 Volcano plot showing differentially expressed genes in K562 shNCOR1 cells compared to K562 shControl cells, specifically examining the expression of genes identified in the cistrome analyses of NCOR1. Red dots show the genes associated with proximal NCOR1 binding (±1 kb of TSS) in combination with either H3K9me3 (Ht), CpG methylation at a low level (Cl) or a high level (Chromatin immunoprecipitation). For each of these analyses the enrichment of genes was tested by a hypergeometric test.
D: NtCh

## Slide 9
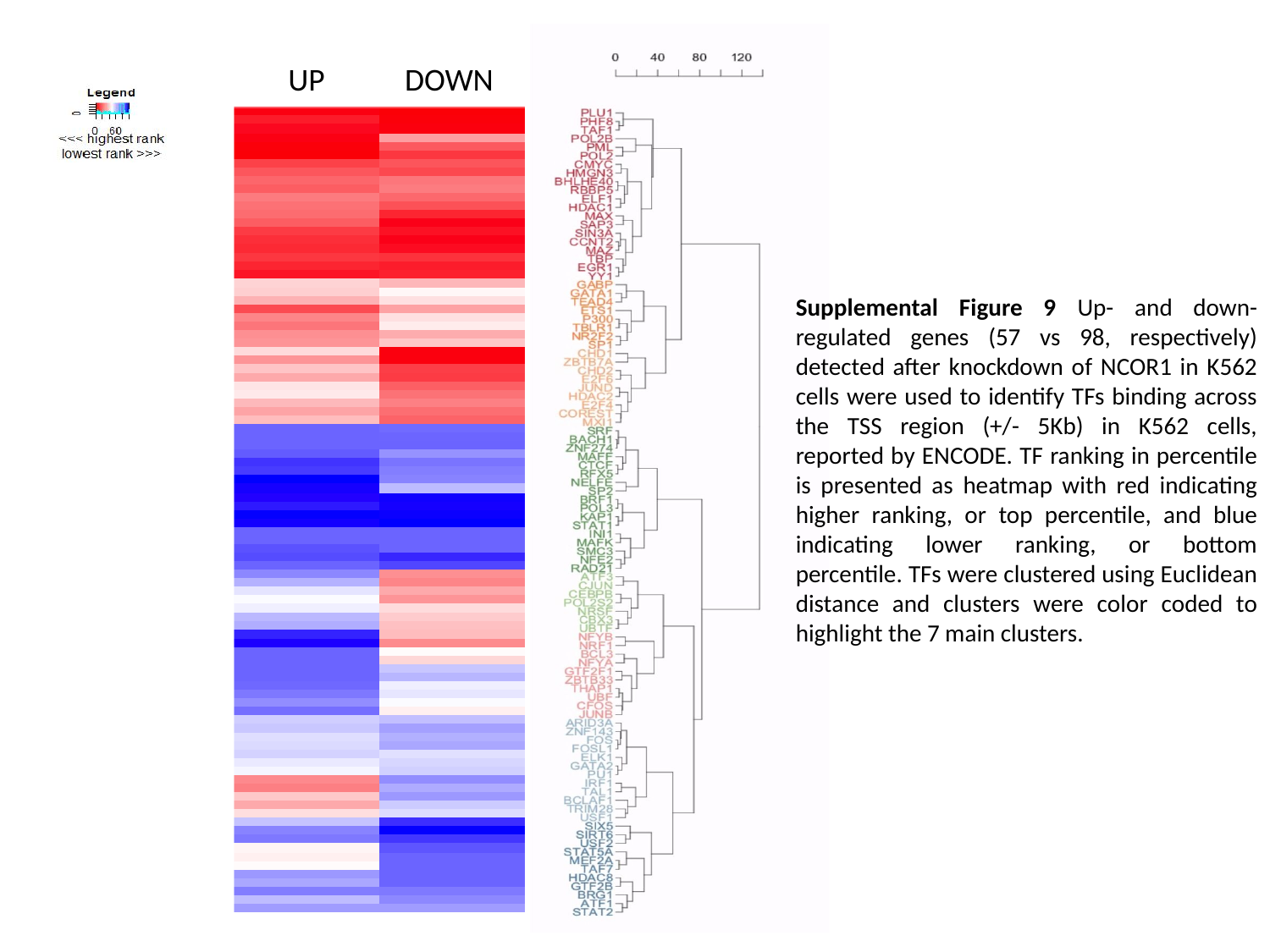

UP
DOWN
Supplemental Figure 9 Up- and down-regulated genes (57 vs 98, respectively) detected after knockdown of NCOR1 in K562 cells were used to identify TFs binding across the TSS region (+/- 5Kb) in K562 cells, reported by ENCODE. TF ranking in percentile is presented as heatmap with red indicating higher ranking, or top percentile, and blue indicating lower ranking, or bottom percentile. TFs were clustered using Euclidean distance and clusters were color coded to highlight the 7 main clusters.

## Slide 10
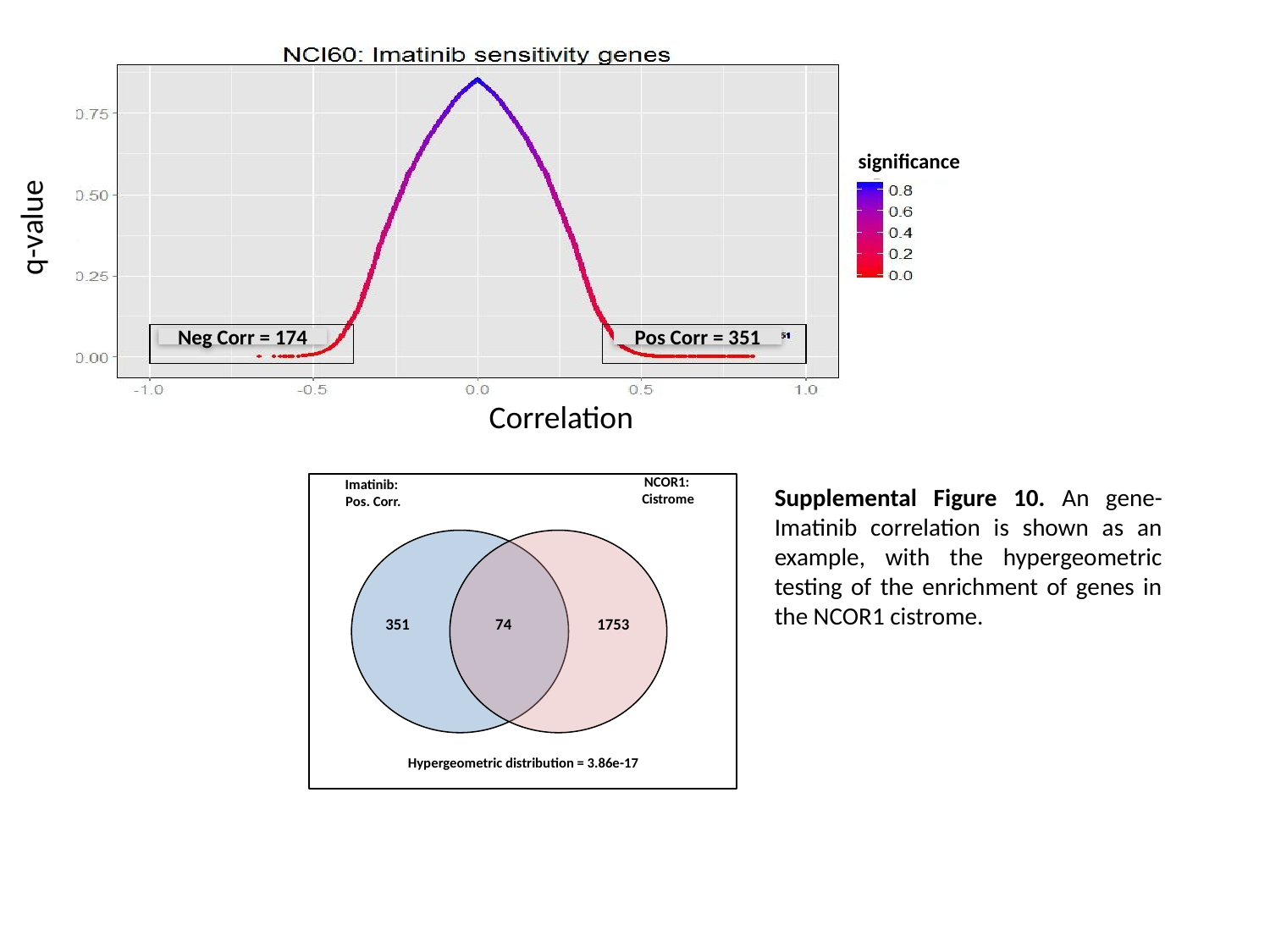

significance
q-value
Neg Corr = 174
Pos Corr = 351
Correlation
NCOR1:
Cistrome
Imatinib:
Pos. Corr.
351
74
1753
Hypergeometric distribution = 3.86e-17
Supplemental Figure 10. An gene-Imatinib correlation is shown as an example, with the hypergeometric testing of the enrichment of genes in the NCOR1 cistrome.

## Slide 11
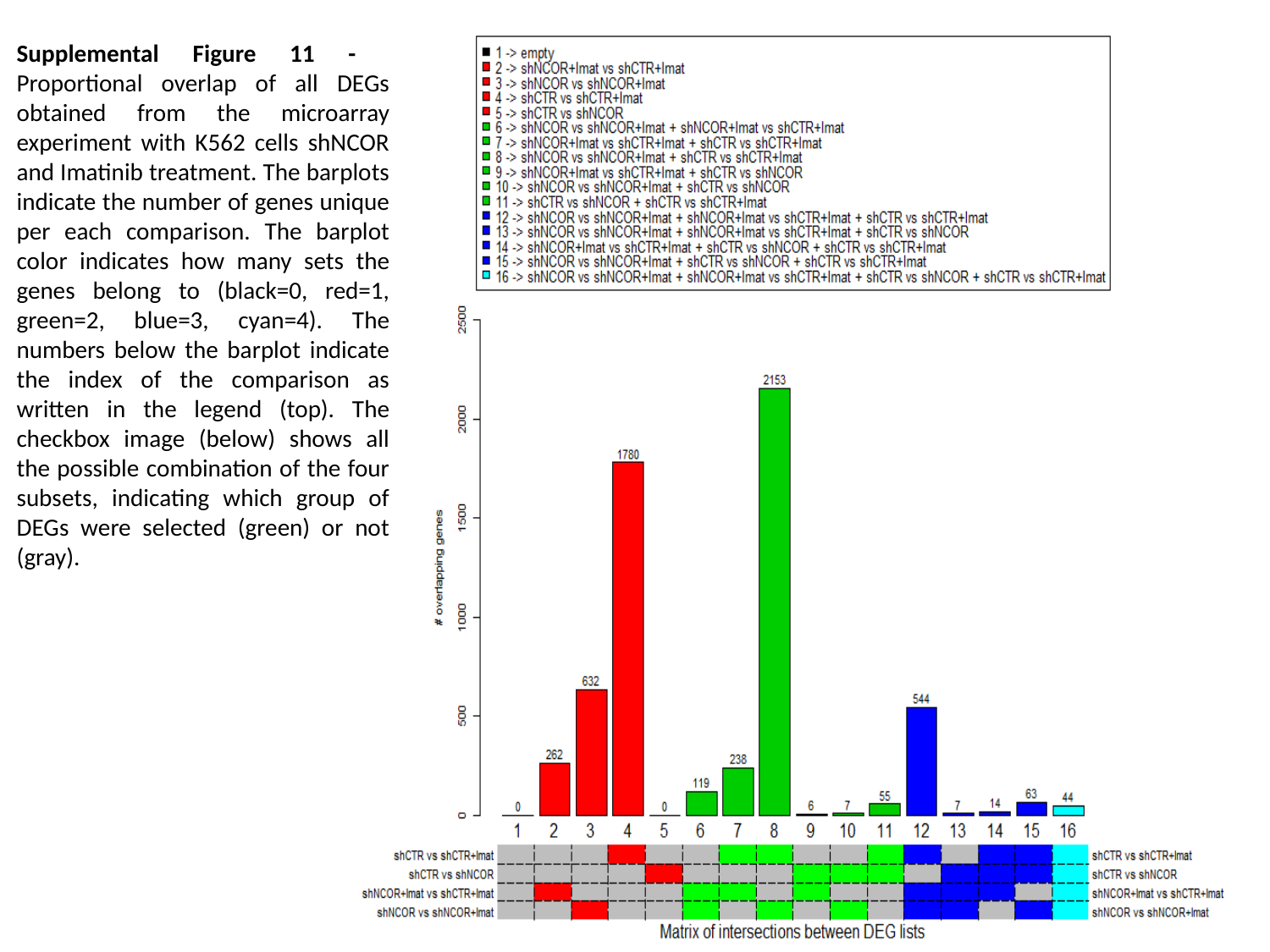

Supplemental Figure 11 - Proportional overlap of all DEGs obtained from the microarray experiment with K562 cells shNCOR and Imatinib treatment. The barplots indicate the number of genes unique per each comparison. The barplot color indicates how many sets the genes belong to (black=0, red=1, green=2, blue=3, cyan=4). The numbers below the barplot indicate the index of the comparison as written in the legend (top). The checkbox image (below) shows all the possible combination of the four subsets, indicating which group of DEGs were selected (green) or not (gray).
